# Comparative Efficacy and Safety of Different Orforglipron Doses in Patients With Type 2 Diabetes Mellitus and Obesity: A Systematic Review and Network Meta-Analysis

**DOI:** 10.7759/cureus.102018

**Published:** 2026-01-21

**Authors:** Marwan Tantoush, Yaseen Almaghrabi, Allaeddin Abusbaeh, Nouraddeen Ahmed, Ayoub Tantush, Bahaeddin Ben Hamida, Malek Sagher, Motasem Sager, Aly Abouslima, Sara E Elbahnasawy, Mahmoud M Elhady, Mohamed Hesham Gamal

**Affiliations:** 1 Internal Medicine, University Hospitals Birmingham NHS Foundation Trust, Birmingham, GBR; 2 Internal Medicine, University of Tripoli, Tripoli, LBY; 3 General Medicine, Heartlands Hospital, University Hospitals Birmingham NHS Foundation Trust, Birmingham, GBR; 4 Geriatrics and General Medicine, Heartlands Hospital, University Hospitals Birmingham NHS Foundation Trust, Birmingham, GBR; 5 General Medicine, University Hospitals Birmingham NHS Foundation Trust, Birmingham, GBR; 6 General Practice, Heartlands Hospital, University Hospitals Birmingham NHS Foundation Trust, Birmingham, GBR; 7 Acute Medicine, Acute Care Common Stem (ACCS) Internal Medicine (IM) Trainee Lead Employer Trust, Newcastle, GBR; 8 Diagnostic Radiology, Menofia University Hospital, Menofia, EGY; 9 Orthopaedics, Faculty of Medicine, Benha University, Al-Qalyubia, EGY; 10 Pharmacy, Banha University Hospitals, Banha, EGY; 11 Pharmacology and Therapeutics, Faculty of Pharmacy, Tanta University, Tanta, EGY

**Keywords:** dose-response relationship, glycemic control, network meta-analysis, obesity, orforglipron, type 2 diabetes mellitus, weight reduction

## Abstract

Type 2 diabetes mellitus (T2DM) and obesity represent major global health challenges. Glucagon-like peptide-1 receptor agonists (GLP-1RAs) have emerged as effective treatments for both conditions, providing significant glycemic control and weight-reduction benefits. Orforglipron is an investigational, oral, non-peptide GLP-1RA that does not require complex absorption enhancers or dosing restrictions. Preclinical and early clinical studies have demonstrated promising results in glycemic control and weight reduction. This systematic review and network meta-analysis aims to evaluate the efficacy and safety of various orforglipron doses in improving glycemic control and reducing body weight.

We conducted a search across five databases. A frequentist network meta-analysis with random-effects models was performed using MetaInsight (version 3.14) to analyze randomized controlled trials(RCTs) comparing orforglipron with placebo in patients with obesity or diabetes. Efficacy outcomes (HbA1c, body weight, BMI, lipid profile, blood pressure) were reported as mean differences, and safety outcomes (adverse events) as risk ratios, with 95% CIs.

Five RCTs involving multiple orforglipron doses (3-45 mg) demonstrated that higher doses, particularly 45 mg, significantly reduced BMI (MD = -3.52 kg/m²), body weight (MD = -9.34%), waist circumference (MD = -7.19 cm), HbA1c (MD = -1.33%), triglycerides (MD = -15.31% for 36 mg), and blood pressure compared to placebo. All doses showed higher rates of total adverse events and treatment discontinuation than placebo, while serious adverse events and specific gastrointestinal symptoms (nausea, vomiting, dyspepsia) were lower than placebo.

Orforglipron is particularly suitable for patients preferring oral therapy over injectable GLP-1 receptor agonists. Orforglipron demonstrated significant dose-dependent improvements in patients with obesity (with or without comorbidities) and T2DM, with higher doses (36-45 mg) showing greater efficacy for weight loss and glycemic control, though at the cost of increased treatment discontinuation. Mid-range doses (24-36 mg) may be better suited for patients prioritizing lipid management and blood pressure control while seeking improved tolerability with a lower discontinuation risk.

## Introduction and background

Diabetes mellitus (DM) affects more than half a billion adults and poses a major challenge to global health systems. Its prevalence continues to rise at an alarming rate, with estimates projecting that the number of affected individuals will exceed 700 million by 2045 [1]. More than 95% of these cases are type 2 DM (T2DM) [2]. This mainly results from a combination of decreased insulin secretion from beta cells and decreased insulin receptor sensitivity [3]. T2DM is strongly associated with obesity and physical inactivity [4].

Obesity is a chronic disease imposing a significant burden on individuals and healthcare systems. Globally, an estimated 2.5 billion people are affected by being overweight or obese; both conditions are closely linked to multiple comorbidities [5]. Obesity increases the risk of developing T2DM, cardiovascular diseases, and certain types of cancer. It can also negatively affect bone health and reproductive function. Moreover, obesity impairs overall quality of life by limiting mobility and disrupting sleep [5]. According to clinical guidelines, pharmacologic therapy for weight management is indicated for individuals with obesity (BMI ≥30 kg/m²) and for those who are overweight with associated comorbid conditions [6,7].

Glucagon-like peptide-1 receptor agonists (GLP-1RAs), incretin-based therapies, are now widely used for the treatment of type 2 diabetes (T2D) and weight reduction [8,9]. GLP-1RAs regulate blood glucose through multiple mechanisms. They stimulate insulin secretion and sensitivity, suppress glucagon release, slow gastric emptying, and reduce appetite. These actions contribute to significantly improved glycemic control and enhanced energy balance [10]. In addition, high-dose GLP-1RAs, particularly semaglutide 2.4 mg and tirzepatide, have demonstrated weight reductions of 15-20% in obesity trials (STEP and SURMOUNT programs), with established cardiovascular benefits in patients with T2DM trials [10,11].

Currently approved GLP-1RAs are peptide-based formulations administered mainly by subcutaneous injection, which may limit patient acceptance and adherence [12,13]. The only approved oral GLP-1RA formulation for T2DM is semaglutide. Due to its peptide nature, it is co-formulated with an absorption enhancer to prevent proteolytic cleavage and facilitate gut uptake [14]. However, it requires strict dosing, such as administration before breakfast under fasting conditions with minimal water intake. Additionally, the currently approved dose (14 mg) has demonstrated modest weight loss relative to injectable semaglutide [15].

A once-daily, oral, non-peptide GLP-1 receptor agonist that is free from dosing restrictions and demonstrates efficacy and tolerability comparable to injectable agents could significantly enhance patient adherence and broaden access to incretin-based therapies [16].

Orforglipron is a novel, chemically synthesized, oral, non-peptide GLP-1RA developed for the treatment of T2DM. Unlike peptide-based agonists, orforglipron does not require co-formulation with complex absorption enhancers [17,18]. It acts as a potent partial agonist, promoting GLP-1R-mediated cyclic adenosine monophosphate (cAMP) accumulation while exerting minimal influence on β-arrestin recruitment [17]. This selectivity demonstrated favorable outcomes across preclinical and clinical evaluations, as β-arrestin is involved in receptor desensitization and endocytosis [19]. In preclinical studies, orforglipron enhanced insulin secretion, decreased food intake in non-human primates, and effectively reduced hyperglycemia in mice expressing the human GLP-1 receptor, but not in receptor knockout models [17]. These data justified the clinical development of orforglipron as an oral, non-peptide GLP-1RA [20].

Although early studies have shown promising results of orforglipron on glycemic control and weight loss, comparative evidence across different doses remains limited. Therefore, this systematic review and meta-analysis aim to assess the efficacy and safety of various orforglipron doses in improving glycemic control and reducing body weight in individuals with T2D and obesity.

## Review

Methods

The methodology and reporting of this systematic review and meta-analysis were guided by the Cochrane Handbook for Systematic Reviews of Interventions and complied with the Preferred Reporting Items for Systematic Reviews and Meta-Analyses (PRISMA) guidelines [21,22].

Search Strategy and Data Collection

PubMed, Web of Science, the Cochrane Library, Embase, and Scopus were systematically searched from database inception to October 23, 2025. The search strategy employed the following terms: (“Orforglipron” OR “LY3502970” OR “LY-3502970” OR “V6G” OR “OWL833”) AND (“Obesity” OR “Obese” OR “Overweight” OR “Adiposity” OR “Corpulence” OR “Plumpness” OR “Excess body weight” OR “Diabetes” OR “Diabetic” OR “MODY” OR “IDDM”). A full detailed search for each database is presented in Appendix 1.

Selection Criteria

We included randomized controlled trials (RCTs) that met the following inclusion criteria: (1) Population: obese or diabetic patients; (2) Intervention: treatment with orforglipron; (3) Comparator: placebo; and (4) Outcomes: studies reporting data on both efficacy and safety endpoints.

Efficacy outcomes included changes in glycated hemoglobin (HbA1c), fasting serum glucose (FSG), body weight (BW), BMI (kg/m²), waist circumference (cm), proportions of participants achieving ≥5%, ≥10%, and ≥15% weight reduction, systolic blood pressure (SBP, mmHg), diastolic blood pressure (DBP, mmHg), total cholesterol (%), high-density lipoprotein (HDL) cholesterol (%), non-HDL cholesterol (%), and triglycerides (%).

Safety outcomes included the incidence of serious adverse events (AEs), total AEs, treatment-discontinuation AEs, and specific AEs, including vomiting, constipation, diarrhea, gastroesophageal reflux disease (GERD), dyspepsia, decreased appetite, eructation, headache, and cardiac disorders. No language restrictions were applied. For studies derived from the same trial registration, we extracted only non-overlapping outcomes to prevent double-counting. Specifically, Rosenstock 2025 and Rosenstock 2025(2) originated from the same Phase 2 trial (NCT05048719); primary efficacy outcomes (HbA1c, BW) were extracted from Rosenstock 2025, while exploratory β-cell function markers were extracted from Rosenstock 2025(2).

Data Extraction

Data extraction was performed using Excel sheets, capturing the following information: (1) summary including study ID, country, study design, orforglipron doses, comparator, study duration, escalation schedule, follow-up period, inclusion criteria, primary endpoint, and conclusion; and (2) baseline characteristics including study arms, age, gender, HbA1c (%) and (mmol/mol), FSG (mg/dL), diabetes duration, BW (kg), metformin use, SBP (mmHg), and DBP (mmHg). Percent change in BW and HbA1c were designated as co-primary outcomes. Secondary outcomes included BMI, waist circumference, FSG, lipid profile parameters (total cholesterol, triglycerides, HDL, LDL), and blood pressure. Data were extracted from the primary endpoint timepoint reported in each trial, ranging from 12 to 72 weeks.

Quality Assessment

We assessed the quality of the included RCTs using Cochrane's risk of bias tool (version 2), as reported in chapter 8.5 of the Cochrane Handbook for Systematic Reviews of Interventions (version 5.1.0). This tool consists of the following assessment items: sequence generation, allocation sequence concealment, blinding of participants and personnel, blinding of outcome assessors, incomplete outcome data, selective outcome reporting, and any other bias. Authors’ judgments for each item were classified into three categories: low, unclear, or high risk of bias. We used the quality assessment table in Part 2, Chapter 8.5 of the same book [23].

Statistical Analysis

A frequentist network meta-analysis with random-effects models was conducted. Continuous outcomes were pooled as mean differences (MDs) with corresponding 95% CIs, while binary outcomes were summarized as risk ratios (RRs). Statistical analyses were conducted using MetaInsight (version 3.14), an interactive web-based application for network meta-analysis implemented in R Shiny and the netmeta package. Heterogeneity was evaluated through clinical, methodological, and statistical domains. Statistical heterogeneity was estimated using the I² statistic [24].

Results

Literature Search

**Figure 1:**
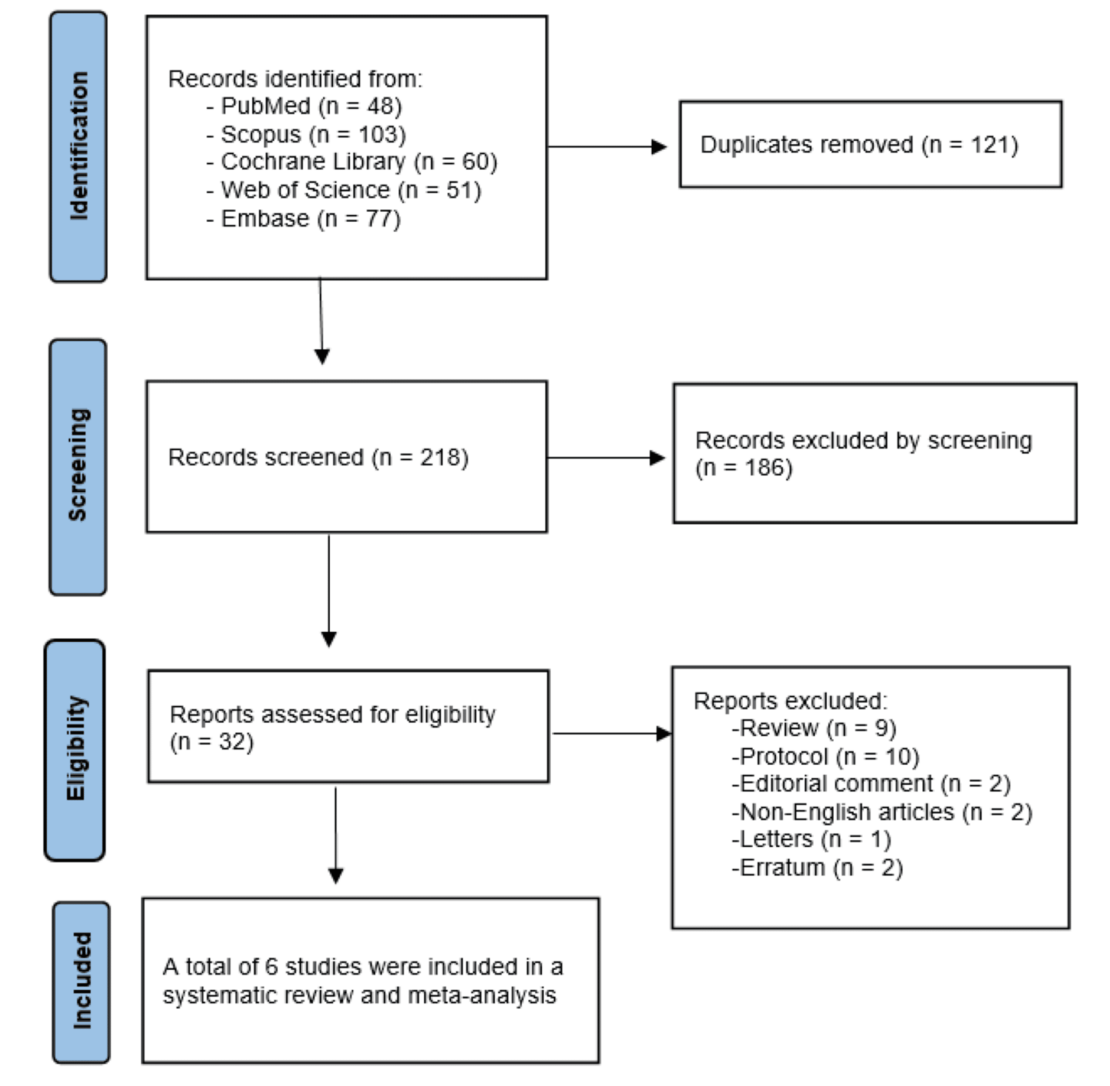
PRISMA flow diagram. References: [11,16,25-28].

Our database search identified 339 records. After removing 121 duplicates, 218 records underwent title and abstract screening. Subsequently, 32 articles were retrieved for full-text evaluation, of which six studies [11,16,25-28] satisfied our eligibility criteria; five studies were included in the analysis, and one study was narrative (Figure 1).

Quality Assessment

**Figure 2:**
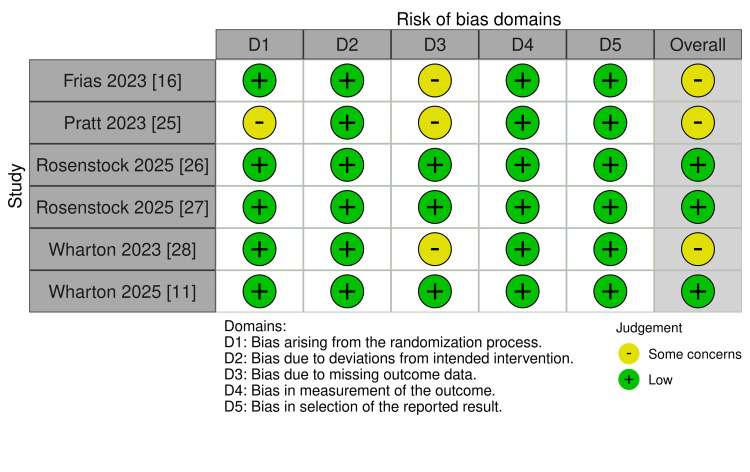
Risk of Bias (ROB 2) assessment. References: [11,16,25-28].

The risk of bias assessment using the ROB2 tool revealed variable quality across the six included RCTs. Three studies demonstrated a low risk of bias across all domains, indicating high methodological quality. The remaining three studies raised concerns, primarily in domains related to randomization (D1) and missing outcome data (D3). All studies demonstrated a low risk of bias in deviations from intended interventions (D2), in the measurement of outcomes (D4), and in the selection of reported results (D5). Overall, three studies were judged to have some concerns about bias, while three studies were considered to have a low risk of bias (Figure 2).

Baseline Characteristics

Baseline characteristics across all studies showed participants with mean ages ranging from 44 to 63 years, male representation varying from 35% to 78%, HbA1c levels between 5.5% and 8.4% in obesity and diabetes studies, respectively, mean BWs spanning 81 kg to 113 kg, BMI ranging from 29 to 39 kg/m², SBP between 114 mmHg and 137 mmHg, and DBP between 78 mmHg and 82 mmHg. Metformin use was reported in 77-100% of participants in diabetes studies. All baseline parameters remained well-balanced across treatment arms within each study (Table 1).

**Table 1:** Baseline characteristics. References: [11,16,25-28] HbA1c: Glycated hemoglobin (hemoglobin A1c); mmol/mol: Millimoles per mole (unit for HbA1c); mg/dL: milligrams per deciliter (unit for fasting serum glucose); kg: kilograms (unit for body weight); BP: Blood pressure; mmHg: millimeters of mercury (unit for blood pressure); n: Number of participants; NA: Not applicable or not available; mg: Milligrams (dose unit for orforglipron).

Study ID	Study arms (n)	Age (years)	Male sex, n (%)	HbA1c (%)	HbA1c (mmol/mol)	Fasting serum glucose (mg/dL)	Diabetes duration (years)	Body weight (kg)	BMI (kg/m²)	Metformin use, n (%)	Systolic BP (mm Hg)	Diastolic BP (mm Hg)
Frias 2023 [16]	Orforglipron 3 mg (n=51)	59.0 ± 9.4	26 (51.0)	8.0 ± 0.8	64.0 ± 9.2	164.0 ± 40.9	5.0 (2.9-11.9)	99.3 ± 25.4	35.3 ± 8.2	44 (86.3)	132.5 ± 11.9	78.3 ± 7.7
	Orforglipron 12 mg (n=56)	57.4 ± 9.2	36 (64.3)	8.2 ± 0.9	66.2 ± 10.1	172.1 ± 42.8	7.1 (3.7-12.6)	99.3 ± 18.1	34.8 ± 6.3	52 (92.9)	134.9 ± 12.6	80.2 ± 8.5
	Orforglipron 24 mg (n=47)	60.5 ± 9.1	30 (63.8)	8.2 ± 0.9	65.6 ± 9.7	171.7 ± 44.4	5.9 (3.3-9.9)	98.5 ± 22.9	34.1 ± 7.7	46 (97.9)	129.7 ± 11.9	78.4 ± 8.8
	Orforglipron 36 mg (n=61)	59.7 ± 9.2	36 (59.0)	8.0 ± 0.7	64.3 ± 8.0	157.9 ± 28.7	5.9 (3.1-9.3)	98.9 ± 17.5	34.4 ± 5.4	54 (88.5)	132.9 ± 12.7	80.5 ± 7.3
	Orforglipron 45 mg (n=63)	58.5 ± 9.4	40 (63.5)	8.1 ± 0.9	65.1 ± 9.6	166.4 ± 35.0	6.8 (2.9-10.6)	104.6 ± 25.1	36.4 ± 6.9	56 (88.9)	136.7 ± 14.0	81.2 ± 8.9
	Placebo (n=55)	58.3 ± 9.5	28 (50.9)	8.1 ± 0.9	64.5 ± 9.5	172.0 ± 42.9	7.8 (4.0-12.5)	102.0 ± 18.8	35.8 ± 6.2	51 (92.7)	135.2 ± 14.6	81.5 ± 7.1
Pratt 2023 [25]	Orforglipron 9 mg (n=9)	57.7 ± 6.4	4 (44.4)	8.02 ± 0.62	64.2 ± 6.8	164.0 ± 40.9	13.48 ± 8.29	85.61 ± 12.76	30.14 ± 3.60	7 (77.8)	132.5 ± 11.9	78.3 ± 7.7
	Orforglipron 15 mg (n=10)	59.6 ± 4.6	7 (70.0)	7.84 ± 0.74	62.2 ± 8.1	172.1 ± 42.8	15.02 ± 11.97	88.02 ± 14.36	30.39 ± 3.61	8 (80.0)	134.9 ± 12.6	80.2 ± 8.5
	Orforglipron 21 mg (n=14)	55.3 ± 8.0	10 (71.4)	8.36 ± 1.31	67.9 ± 14.3	171.7 ± 44.4	9.48 ± 5.48	92.09 ± 18.78	32.60 ± 5.48	14 (100)	129.7 ± 11.9	78.4 ± 8.8
	Orforglipron 27 mg (n=9)	58.8 ± 4.6	7 (77.8)	7.82 ± 0.69	62.0 ± 7.5	157.9 ± 28.7	7.60 ± 4.39	92.80 ± 15.36	30.62 ± 3.55	8 (88.9)	132.9 ± 12.7	80.5 ± 7.3
	Orforglipron 45 mg (n=9)	62.8 ± 4.4	4 (44.4)	7.93 ± 0.79	63.2 ± 8.6	166.4 ± 35.0	10.38 ± 4.78	81.49 ± 10.24	29.82 ± 2.84	9 (100)	136.7 ± 14.0	81.2 ± 8.9
	Placebo (n=17)	56.0 ± 6.0	10 (58.8)	8.09 ± 0.75	64.9 ± 8.2	172.0 ± 42.9	8.63 ± 4.89	90.29 ± 20.04	31.31 ± 4.86	15 (88.2)	135.2 ± 14.6	81.5 ± 7.1
Rosenstock 2025 [26]	Orforglipron 3 mg (n=143)	53.3 ± 11.3	80 (55.9)	7.93 ± 0.86	63.2 ± 9.4	142.9 ± 38.7	4.0 ± 4.8	90.3 ± 25.7	32.9 ± 8.0	NA	126.5 ± 14.1	79.9 ± 9.5
	Orforglipron 12 mg (n=137)	54.1 ± 11.8	66 (48.2)	7.98 ± 0.91	63.8 ± 9.9	155.3 ± 55.1	5.1 ± 6.0	90.6 ± 23.1	33.3 ± 7.8	NA	127.5 ± 14.5	80.5 ± 9.3
	Orforglipron 36 mg (n=141)	52.8 ± 11.8	69 (48.9)	8.07 ± 0.90	64.7 ± 9.8	148.8 ± 40.0	4.2 ± 5.1	90.1 ± 22.9	33.1 ± 7.3	NA	128.3 ± 14.3	80.8 ± 9.1
	Placebo (n=138)	53.3 ± 12.5	75 (54.3)	7.96 ± 0.89	63.8 ± 9.7	143.3 ± 42.2	4.4 ± 5.6	90.0 ± 20.7	32.9 ± 6.8	NA	128.4 ± 14.1	80.1 ± 8.6
Rosenstock 2025 (2) [27]	Orforglipron 3 mg (n=50)	58.7 ± 9.3	26 (48.0)	8.0 ± 0.8	64.0 ± 9.2	164.0 ± 40.9	7.2 ± 6.2	98.6 ± 25.2	34.9 ± 7.7	44 (86.3)	132.5 ± 11.9	78.3 ± 7.7
	Orforglipron 12 mg (n=54)	57.7 ± 9.1	19 (35.2)	8.2 ± 0.9	66.2 ± 10.1	172.1 ± 42.8	8.9 ± 7.7	99.8 ± 17.7	35.0 ± 6.2	52 (92.9)	134.9 ± 12.6	80.2 ± 8.5
	Orforglipron 24 mg (n=46)	60.5 ± 9.2	17 (37.0)	8.2 ± 0.9	65.6 ± 9.7	171.7 ± 44.4	6.8 ± 4.4	98.3 ± 23.1	34.0 ± 7.8	46 (97.9)	129.7 ± 11.9	78.4 ± 8.8
	Orforglipron 36 mg (n=60)	59.6 ± 9.2	25 (41.7)	8.0 ± 0.7	64.3 ± 8.0	157.9 ± 28.7	7.0 ± 5.3	98.9 ± 17.7	34.5 ± 5.5	54 (88.5)	132.9 ± 12.7	80.5 ± 7.3
	Orforglipron 45 mg (n=63)	58.5 ± 9.4	23 (36.5)	8.1 ± 0.9	65.1 ± 9.6	166.4 ± 35.0	8.3 ± 7.3	104.6 ± 25.1	36.4 ± 6.9	56 (88.9)	136.7 ± 14.0	81.2 ± 8.9
	Dulaglutide 1.5 mg (n=50)	58.8 ± 10.2	20 (40.0)	8.0 ± 0.7	64.5 ± 9.5	172.0 ± 42.9	10.2 ± 8.4	98.8 ± 22.1	35.4 ± 8.0	51 (92.7)	114.0 ± 16.4	81.5 ± 7.1
	Placebo (n=55)	58.3 ± 9.5	27 (49.1)	8.1 ± 0.9	64.5 ± 9.5	172.0 ± 42.9	8.6 ± 5.8	102.0 ± 18.8	35.8 ± 6.2	51 (92.7)	135.2 ± 14.6	81.5 ± 7.1
Wharton 2023 [28]	Orforglipron 12 mg (n=50)	49.8 ± 10.5	19 (38)	5.5 ± 0.4	NA	94.4 ± 9.8	NA	107.5 ± 25.3	37.7 ± 7.7	NA	129.4 ± 12.1	82.9 ± 6.8
	Orforglipron 24 mg (n=53)	57.0 ± 9.1	23 (43)	5.7 ± 0.3	NA	97.5 ± 12.0	NA	112.1 ± 30.2	38.1 ± 7.7	NA	129.7 ± 10.8	82.1 ± 7.4
	Orforglipron 36 mg, Subcohort 1 (n=29)	56.3 ± 11.8	11 (38)	5.7 ± 0.4	NA	95.7 ± 13.2	NA	107.8 ± 22.5	38.0 ± 6.4	NA	131.1 ± 11.4	81.5 ± 7.6
	Orforglipron 36 mg, Subcohort 2 (n=29)	55.4 ± 10.9	11 (38)	5.6 ± 0.4	NA	98.0 ± 13.5	NA	108.8 ± 28.5	38.0 ± 6.3	NA	131.7 ± 12.6	81.2 ± 8.1
	Orforglipron 45 mg, Subcohort 1 (n=31)	56.5 ± 10.7	12 (39)	5.7 ± 0.3	NA	98.0 ± 8.5	NA	105.2 ± 20.4	36.8 ± 5.5	NA	128.9 ± 11.0	80.6 ± 8.5
	Orforglipron 45 mg, Subcohort 2 (n=30)	50.9 ± 12.6	14 (47)	5.6 ± 0.4	NA	92.3 ± 10.1	NA	110.9 ± 28.1	38.7 ± 7.6	NA	126.4 ± 11.6	78.0 ± 8.5
	Placebo (n=50)	54.0 ± 8.8	21 (42)	5.6 ± 0.4	NA	97.2 ± 10.2	NA	107.6 ± 25.2	37.8 ± 6.5	NA	128.5 ± 9.5	81.5 ± 7.2
Wharton 2025 [11]	Orforglipron 6 mg (n=723)	44.9 ± 12.1	254 (35.1)	NA	NA	NA	NA	103.2 ± 21.7	37.0 ± 6.5	NA	125.4 ± 14.1	81.0 ± 9.3
	Orforglipron 12 mg (n=725)	45.4 ± 12.6	258 (35.6)	NA	NA	NA	NA	102.2 ± 21.6	36.7 ± 6.5	NA	125.1 ± 13.7	81.2 ± 9.4
	Orforglipron 36 mg (n=730)	44.9 ± 11.9	265 (36.3)	NA	NA	NA	NA	103.1 ± 23.2	36.9 ± 6.7	NA	125.8 ± 15.9	80.9 ± 10.1
	Placebo (n=949)	45.1 ± 11.9	341 (35.9)	NA	NA	NA	NA	103.9 ± 22.0	37.1 ± 6.3	NA	125.8 ± 14.5	81.8 ± 9.9

Summary of the Included Studies

All six included studies were RCTs conducted across multiple countries (USA, Canada, Mexico, Japan, China, India, Hungary, Poland, Slovakia, Saudi Arabia, Spain, and Brazil). The doses ranged from 3 mg to 45 mg compared with placebo or dulaglutide 1.5 mg. Study durations varied from 12 to 72 weeks. The studies employed different dose escalation schedules in participants with either type 2 diabetes inadequately controlled on diet and exercise with or without metformin, or obesity without diabetes (Table 2).

**Table 2:** Summary of the included studies. References: [11,16,25-28] HbA1c: Glycated hemoglobin (hemoglobin A1c); kg/m²: Kilograms per square meter (unit of BMI); GLP-1: Glucagon-like peptide-1; T2D: Type 2 diabetes; USA: United States of America; NCT: ClinicalTrials.gov identifier prefix; mg: Milligrams; HOMA-B: Homeostatic model assessment of β-cell function; HOMA-IR: Homeostatic model assessment of insulin resistance; IGFBP2: Insulin-like growth factor binding protein 2.

Study ID	Study design	Registration	Orforglipron doses	Comparator	Study duration	Escalation schedule	Follow-up period	Countries	Inclusion criteria	Primary endpoints	Conclusion
Frias 2023 [16]	Phase 2, multicentre, randomised, double-blind, dose-response	NCT05048719	3 mg, 12 mg, 24 mg, 36 mg (2 groups), 45 mg (2 groups)	Placebo; dulaglutide 1.5 mg once weekly	26 weeks	Two different dose-escalation regimens for each of the 36 mg and 45 mg cohorts	26 weeks treatment + 2-week safety follow-up	USA, Hungary, Poland, Slovakia	Adults ≥18 years with T2D treated with diet and exercise with or without stable metformin (≥3 months). HbA1c 7.0-10.5%, BMI ≥23 kg/m², stable body weight (≤5% change) for 3 months before randomisation	Change in HbA1c from baseline to week 26 (orforglipron vs placebo)	In this phase 2 trial the novel, oral, non-peptide GLP-1 receptor agonist orforglipron at doses of 12 mg or greater showed significant reductions in HbA1c and bodyweight compared with placebo or dulaglutide. The adverse event profile was similar to other GLP-1 receptor agonists in similar stage of development. Orforglipron might provide an alternative to injectable GLP-1 receptor agonists and oral semaglutide, with the prospect of less burdensome administration to achieve treatment goals in people with type 2 diabetes.
Pratt 2023 [25]	Phase 1b, multicentre, double-blind, placebo-controlled, multiple-ascending-dose	NCT04426474	9 mg, 15 mg, 21 mg, 27 mg, 45 mg	Placebo; dulaglutide 1.5 mg weekly	12 weeks	Weekly escalation	12 weeks treatment	USA, Germany	Aged 18-70 years (18-64 in Germany) with T2D for ≥6 months, treated with diet/exercise alone or stable metformin for ≥3 months. HbA1c 7.0-10.5% (7.0-9.0% in Germany), BMI 18.5-45 kg/m² (18.5-35 kg/m² in Germany), stable body weight (<5% change) for 3 months. No other glucose-lowering meds within 3 months; no prior insulin therapy	Safety and tolerability	Orforglipron treatment resulted in meaningful reductions in HbA1c and body weight, with an adverse event profile consistent with that of other GLP-1RAs. Orforglipron may provide a safe and effective once-daily oral treatment alternative to injectable GLP-1RAs or peptide oral formulations without water and food restrictions.
Rosenstock 2025 [26]	Phase 3, multicentre, double-blind, randomised, placebo-controlled	NCT05971940	3 mg, 12 mg, 36 mg	Placebo	40 weeks	Every 4 weeks	40 weeks treatment + 2-week safety follow-up	USA, Mexico, Japan, India, China	≥18 years with T2D inadequately controlled with diet and exercise alone. HbA1c 7.0-9.5%, BMI ≥23 kg/m², stable weight (within 5%) for 3 months. No glucose-lowering meds within 3 months; never received insulin	Change in HbA1c from baseline to week 40	In adults with early type 2 diabetes, orforglipron significantly reduced the glycated hemoglobin level over a period of 40 weeks.
Rosenstock 2025 (2) [27]	Phase 2, multicentre, randomised, double-blind, dose-response trial (participant-level exploratory analyses)	NCT05048719	3 mg, 12 mg, 24 mg, 36 mg, 45 mg	Dulaglutide 1.5 mg once weekly (SC); placebo	26 weeks	The 36 mg and 45 mg maintenance doses each had two different dose-escalation schedules	26 weeks treatment	USA, Hungary, Poland, Slovakia	Inadequately controlled T2D randomly assigned to orforglipron (3, 12, 24, 36, or 45 mg), dulaglutide (1.5 mg), or placebo	Treatment effects on β-cell function and insulin sensitivity markers, including HOMA-B and HOMA-IR, fasting C-peptide, insulin, serum glucose, glucagon, adiponectin, IGFBP2, proinsulin, and proinsulin/insulin ratio	Increased HOMA-B and improved metabolic biomarkers suggest that treatment with the orally administered non-peptide GLP-1 receptor agonist orforglipron enhanced pancreatic β-cell function and insulin sensitivity in patients with type 2 diabetes.
Wharton 2023 [28]	Phase 2, randomised, double-blind, placebo-controlled trial	NCT05051579	12 mg, 24 mg, 36 mg (2 subcohorts), 45 mg (2 subcohorts)	Placebo	36 weeks	Escalation done every two weeks	36 weeks treatment + 2-week safety follow-up	Canada, USA, Hungary	Adults (18-75 years) with obesity (BMI ≥30) or overweight (BMI 27 to <30) plus ≥1 weight-related coexisting condition; without diabetes (HbA1c <6.5%); stable body weight (≤5% gain/loss for 3 months before randomisation)	Percentage change from baseline in body weight at week 26	Daily oral orforglipron, a nonpeptide GLP-1 receptor agonist, was associated with weight reduction. Adverse events reported with orforglipron were similar to those with injectable GLP-1 receptor agonists.
Wharton 2025 [11]	Phase 3, multinational, randomised, double-blind, placebo-controlled, parallel-group trial	NCT05869903	6 mg, 12 mg, 36 mg	Placebo	72 weeks	6 mg maintenance reached at week 8; 12 mg maintenance reached at week 12; 36 mg maintenance reached at week 20	72 weeks treatment + 2-week off-drug safety follow-up	9 countries: USA, Canada, Saudi Arabia, Spain, Japan, Brazil, Hungary, China, Mexico	Adults ≥18 years with BMI ≥30 OR BMI 27-30 with ≥1 obesity-related complication; ≥1 prior unsuccessful dietary effort; no diabetes (HbA1c <6.5%); stable body weight (≤5 kg change within 90 days before screening)	Percent change in body weight from baseline to week 72	In adults with obesity, 72-week treatment with orforglipron led to significantly greater reductions in body weight than placebo; the adverse-event profile was consistent with that of other GLP-1 receptor agonists.

Weight Outcomes

**Figure 3:**
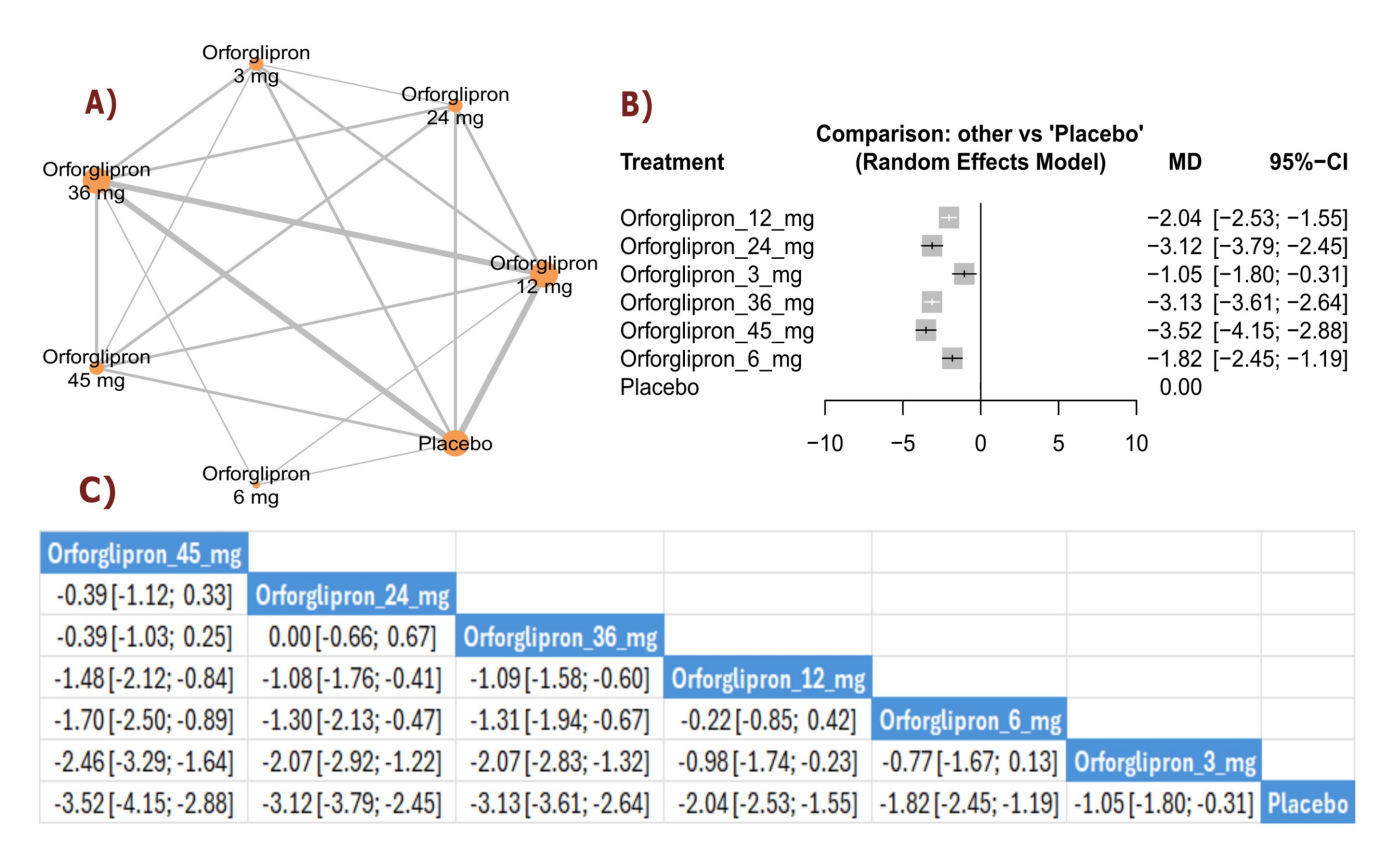
BMI change (kg/m²). References: [11,16,25,26,28].

BMI change (kg/m²): The results showed a significant reduction in BMI with orforglipron 45 mg compared with orforglipron 12 mg (MD = -1.48, 95% CI (-2.12; -0.84)), in addition to a significant reduction between orforglipron 45 mg and orforglipron 3 mg (MD = -2.46, 95% CI (-3.29; -1.64)). Most doses demonstrated a statistically significant reduction in BMI compared with placebo, with orforglipron 45 mg showing the most significant reduction versus placebo (MD = -3.52, 95% CI (-4.15; -2.88)). The top three treatments for BMI reduction, ranked by P-score, were the 45 mg dose, followed by the 24 mg dose, and the 36 mg dose (Figure 3).

**Figure 4:**
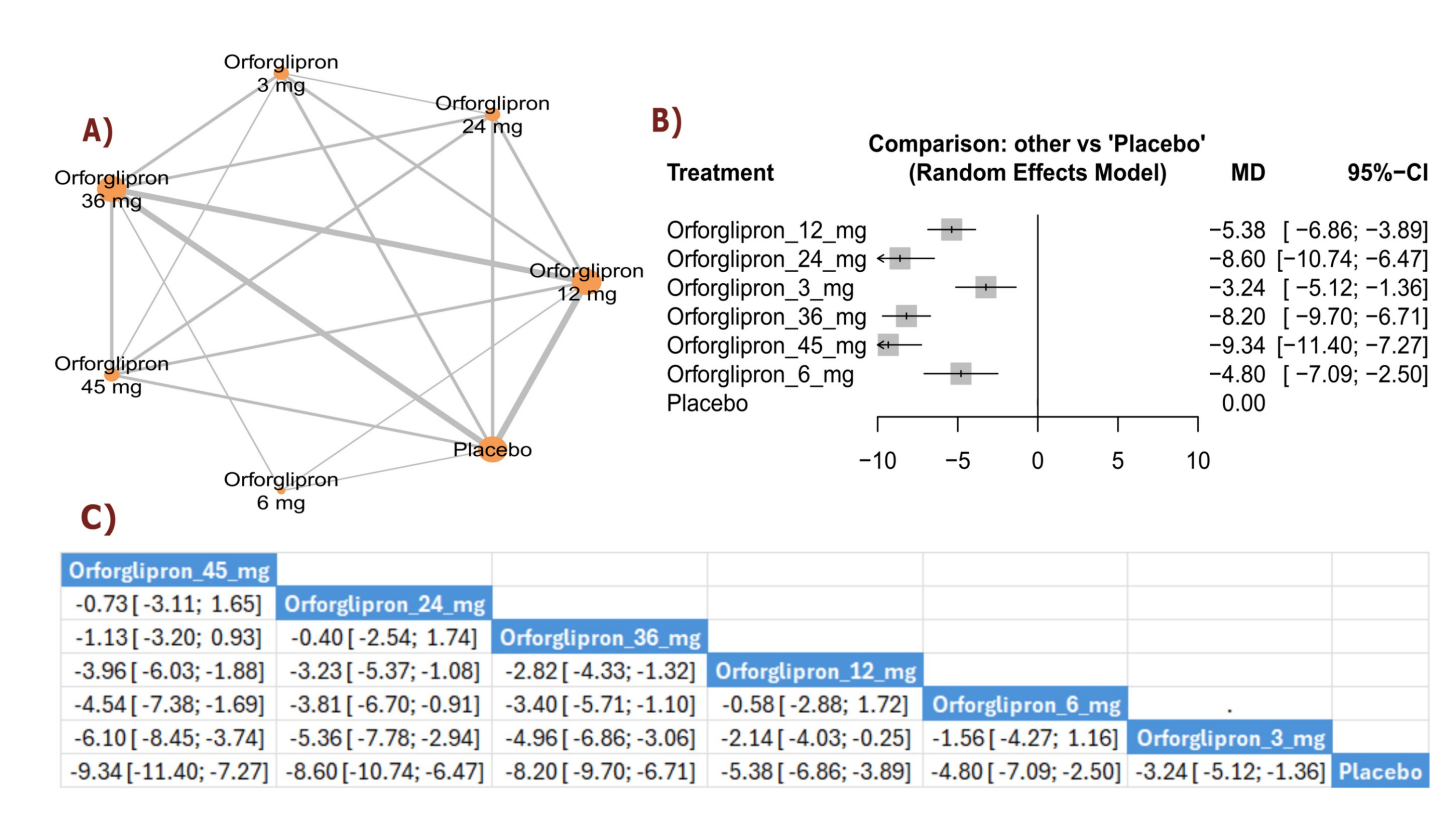
BMI change (%). References: [11,16,25,26,28].

BW change (percent): The results demonstrated a significant reduction in BW percentage between orforglipron 45 mg and orforglipron 12 mg (MD = -3.96, 95% CI (-6.03; -1.88)), as well as a significant reduction between orforglipron 45 mg and orforglipron 3 mg (MD = -6.10, 95% CI (-8.45; -3.74)). All orforglipron doses showed significant decreases compared with placebo, with the 45 mg dose achieving the most significant effect (MD = -9.34, 95% CI (-11.40; -7.27)). The top three treatments for BW percentage reduction according to P-score were the 45 mg dose, followed by the 24 mg dose, and the 36 mg dose (Figure 4).

**Figure 5:**
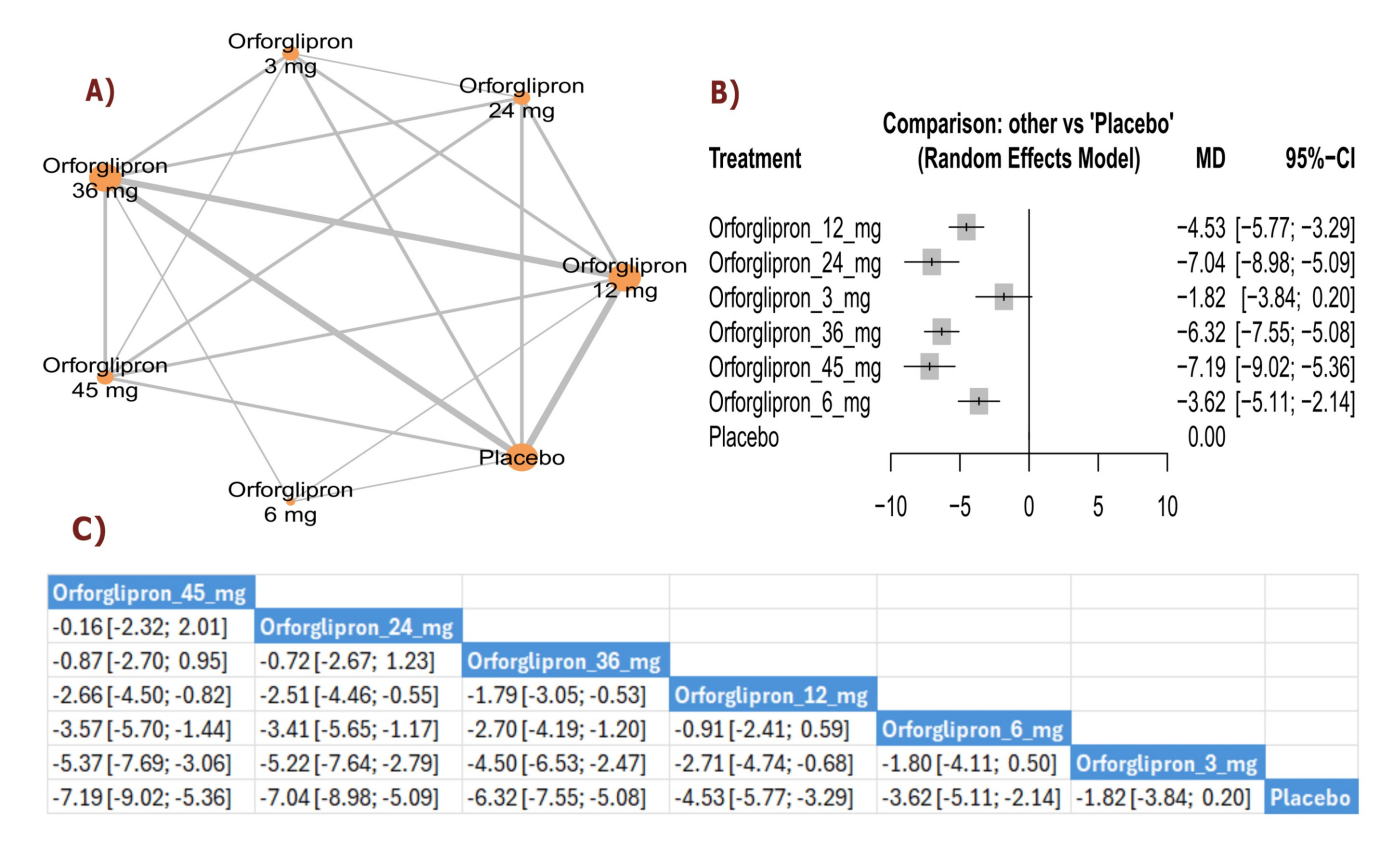
Waist circumference change (cm). References: [11,16,25,26,28].

Waist circumference change (cm): The results showed a significant reduction in waist circumference between orforglipron 45 mg and orforglipron 12 mg (MD = -2.66, 95% CI (-4.50; -0.82)), in addition to a significant reduction between orforglipron 45 mg and orforglipron 3 mg (MD = -5.37, 95% CI (-7.69; -3.06)). Most higher doses demonstrated significant reductions compared with placebo, with orforglipron 45 mg showing the most significant reduction versus placebo (MD = -7.19, 95% CI (-9.02; -5.36)). The top three treatments for waist circumference reduction, ranked by P-score, were orforglipron 45 mg, orforglipron 24 mg, and orforglipron 36 mg (Figure 5).

**Figure 6:**
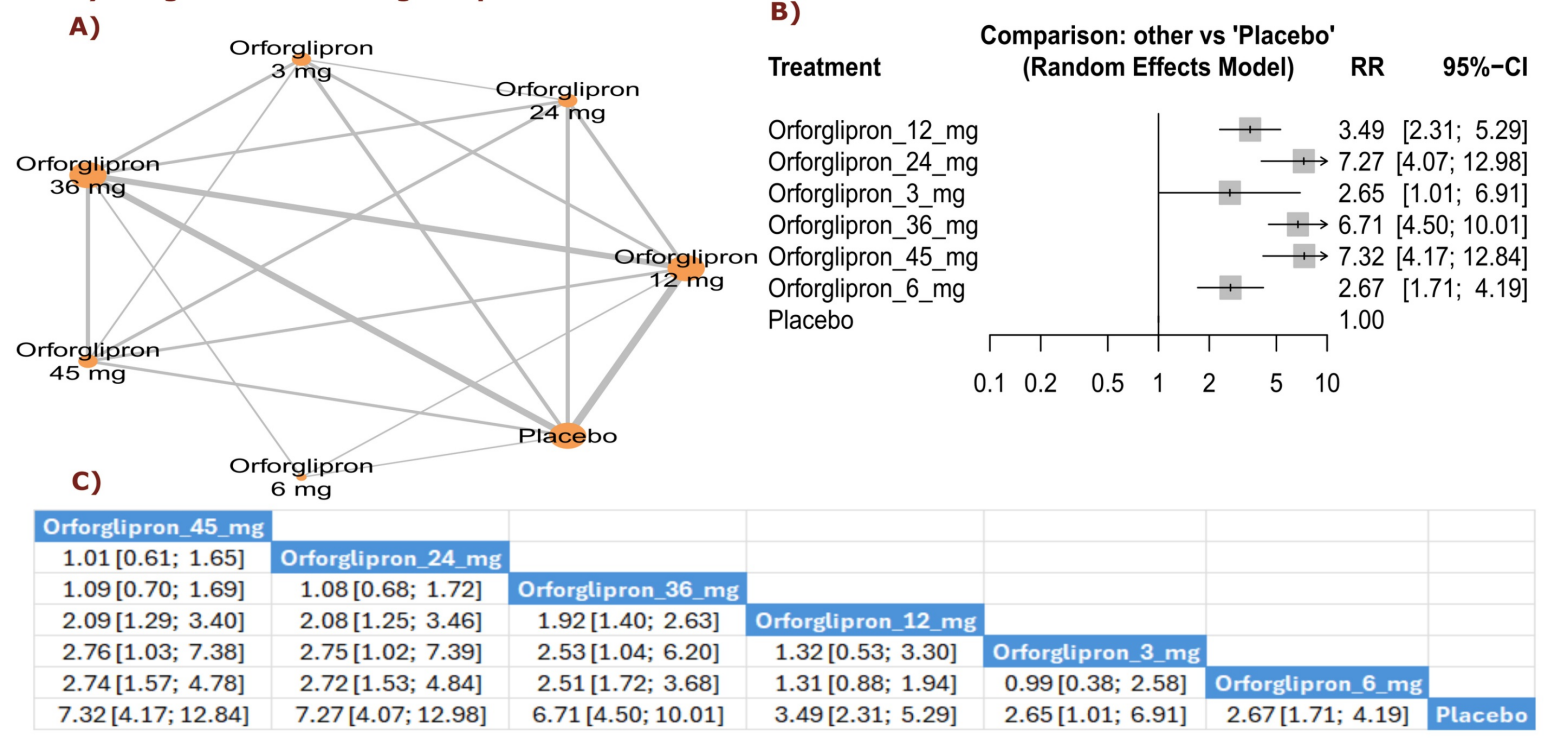
Weight reduction ≥15%. References: [11,16,25,26,28].

Weight reduction ≥15%: The results demonstrated a significant increase in achieving ≥15% weight reduction with orforglipron 45 mg compared with orforglipron 12 mg (RR = 2.09, 95% CI (1.29; 3.40)), as well as between orforglipron 45 mg and orforglipron 3 mg (RR = 2.76, 95% CI (1.03; 7.38)). All orforglipron doses showed significantly higher rates of achieving ≥15% weight reduction than placebo, with the 45 mg dose demonstrating the most significant effect (RR = 7.32, 95% CI (4.17; 12.84)). The top three treatments for achieving weight reduction ≥15% according to P-score were orforglipron 45 mg, followed by orforglipron 24 mg, and orforglipron 36 mg (Figure 6).

Glycemic Outcomes

**Figure 7:**
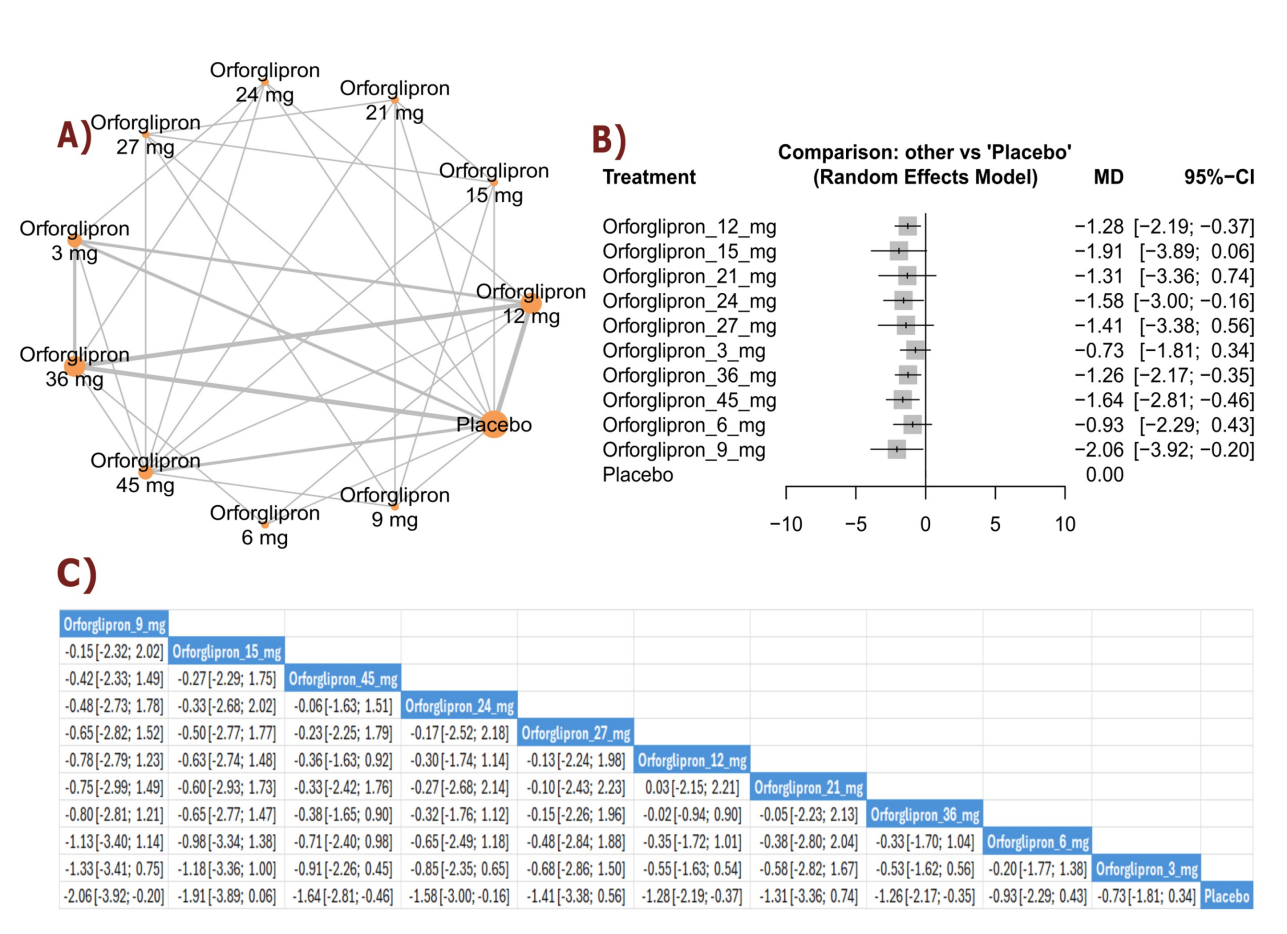
Fasting serum glucose change (mmol/L). References: [11,16,25,26,28].

FSG change (mmol/L): The results showed a significant reduction in FSG with orforglipron 9 mg compared with placebo (MD = -2.06, 95% CI (-3.92; -0.20)). Multiple orforglipron doses demonstrated significant reductions in fasting glucose compared with placebo. The top three treatments for FSG reduction, ranked by P-score, were orforglipron 9 mg, orforglipron 15 mg, and orforglipron 45 mg (Figure 7).

**Figure 8:**
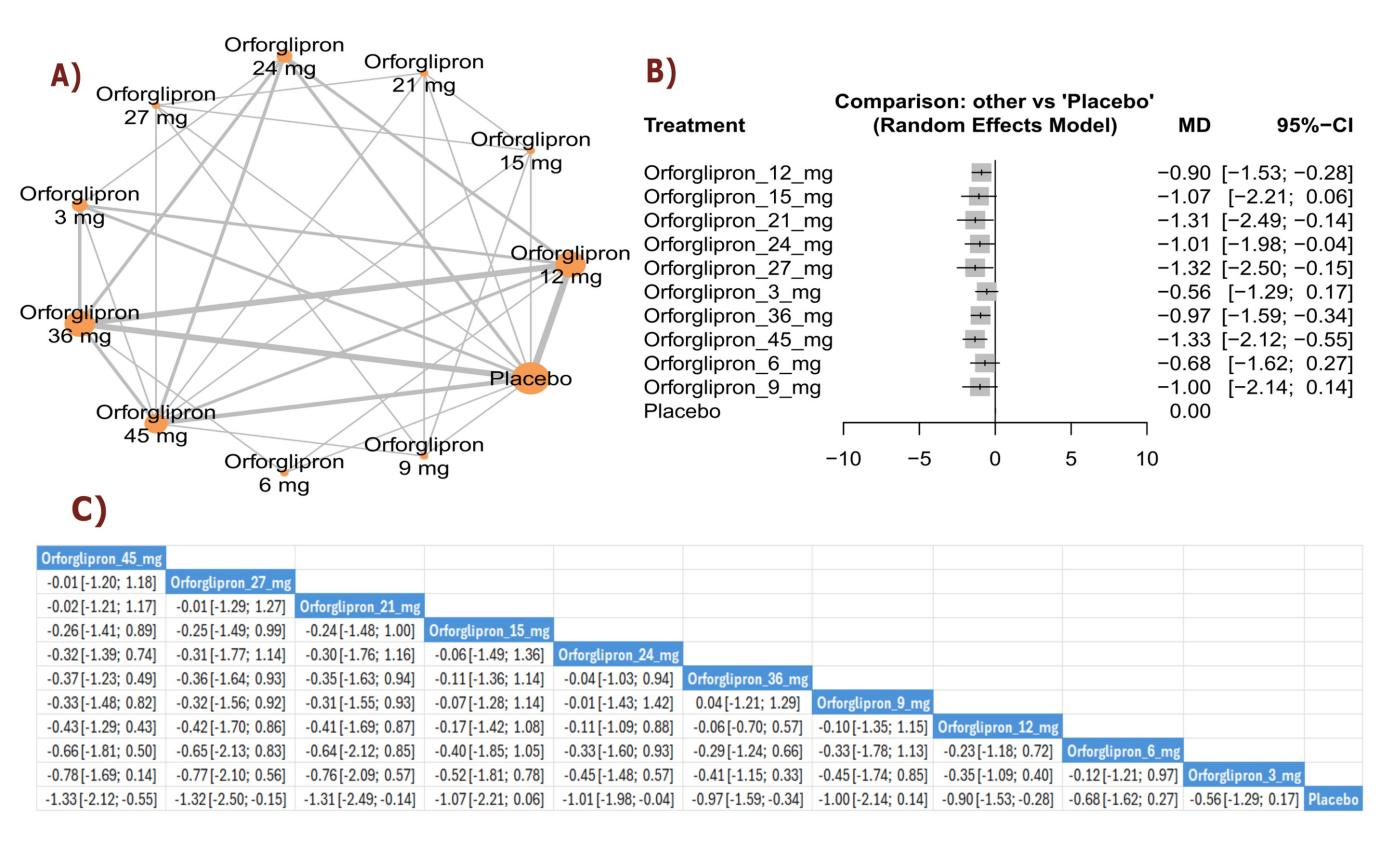
HbA1c change (%). References: [11,16,25,26,28].

HbA1c change (percent): The results demonstrated a significant reduction in HbA1c with orforglipron 45 mg compared with placebo (MD = -1.33, 95% CI (-2.12; -0.55)), as well as with orforglipron 24 mg compared with placebo (MD = -1.01, 95% CI (-1.98; -0.04)). Most orforglipron doses showed significant reductions in HbA1c compared with placebo. The top three treatments for HbA1c reduction, ranked by P-score, were orforglipron 45 mg, orforglipron 27 mg, and orforglipron 21 mg (Figure 8).

Lipid Profile Outcomes

**Figure 9:**
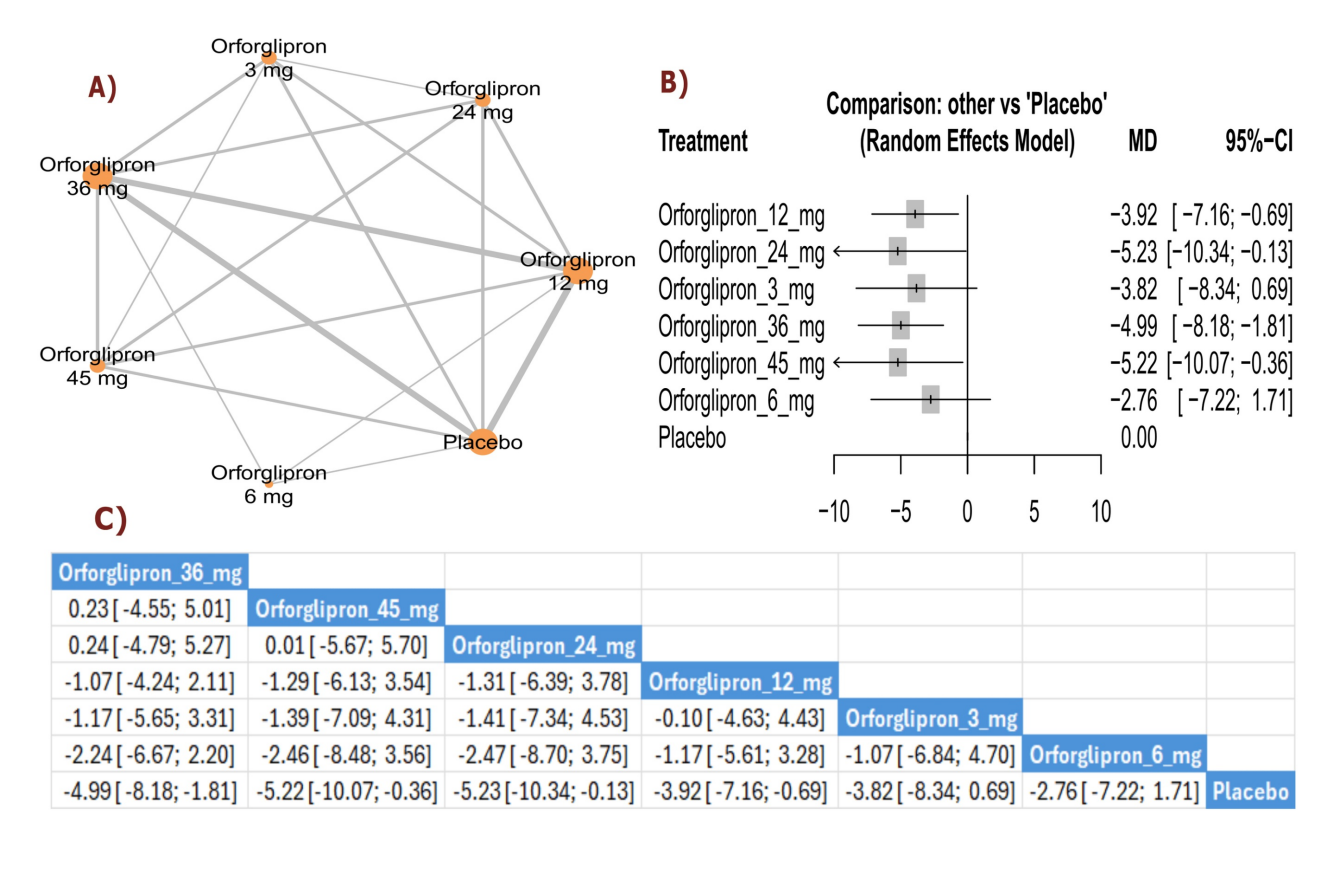
Total cholesterol change (%). References: [11,16,25,26,28].

Total cholesterol change (percent): The results showed a significant reduction in total cholesterol with orforglipron 36 mg compared with placebo (MD = -4.99, 95% CI (-8.18; -1.81)). The top three treatments for total cholesterol reduction, ranked by P-score, were orforglipron 36 mg, orforglipron 45 mg, and orforglipron 24 mg (Figure 9).

**Figure 10:**
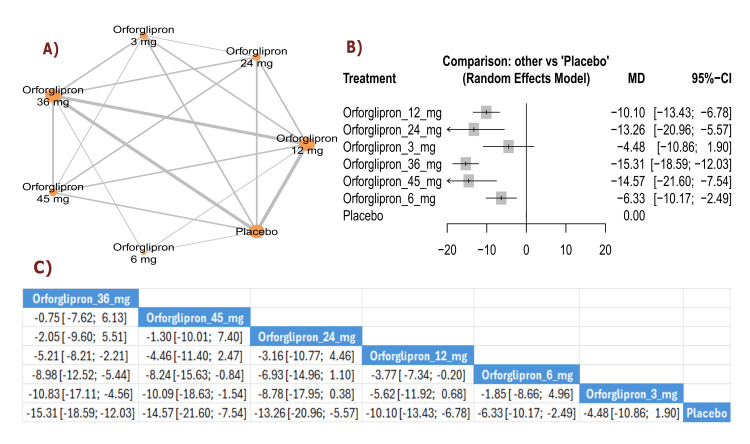
Triglycerides change (%). References: [11,16,25,26,28].

Triglycerides change (percent): The results demonstrated a significant reduction in triglycerides between orforglipron 36 mg and orforglipron 12 mg (MD = -5.21, 95% CI (-8.21; -2.21)), in addition to a significant reduction between orforglipron 36 mg and orforglipron 6 mg (MD = -8.98, 95% CI (-12.52; -5.44)). All orforglipron doses showed significant triglyceride reductions compared with placebo, with orforglipron 36 mg demonstrating the most significant effect (MD = -15.31, 95% CI (-18.59; -12.03)). The top three treatments for triglyceride reduction, ranked by P-score, were orforglipron 36 mg, orforglipron 45 mg, and orforglipron 24 mg (Figure 10).

**Figure 11:**
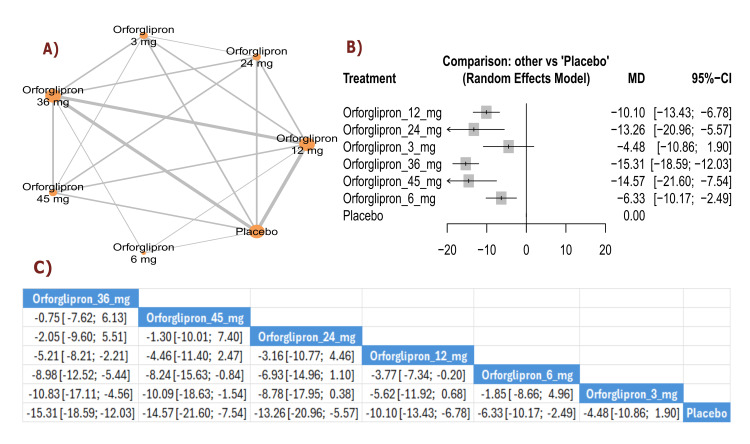
Triglycerides change (%). References: [11,16,25,26,28].

HDL cholesterol change (percent): The results showed a significant reduction (worsening) in HDL cholesterol between placebo and orforglipron 6 mg (MD = -2.55, 95% CI (-4.48; -0.62)), as well as between placebo and orforglipron 45 mg (MD = -3.17, 95% CI (-6.17; -0.17)). Most orforglipron doses demonstrated significant reductions in HDL cholesterol compared with placebo, except for the 3 mg dose. The top three treatments for HDL cholesterol preservation, ranked by P-score, were the 36 mg dose, followed by the 24 mg dose, and the 12 mg dose (Figure 11).

**Figure 12:**
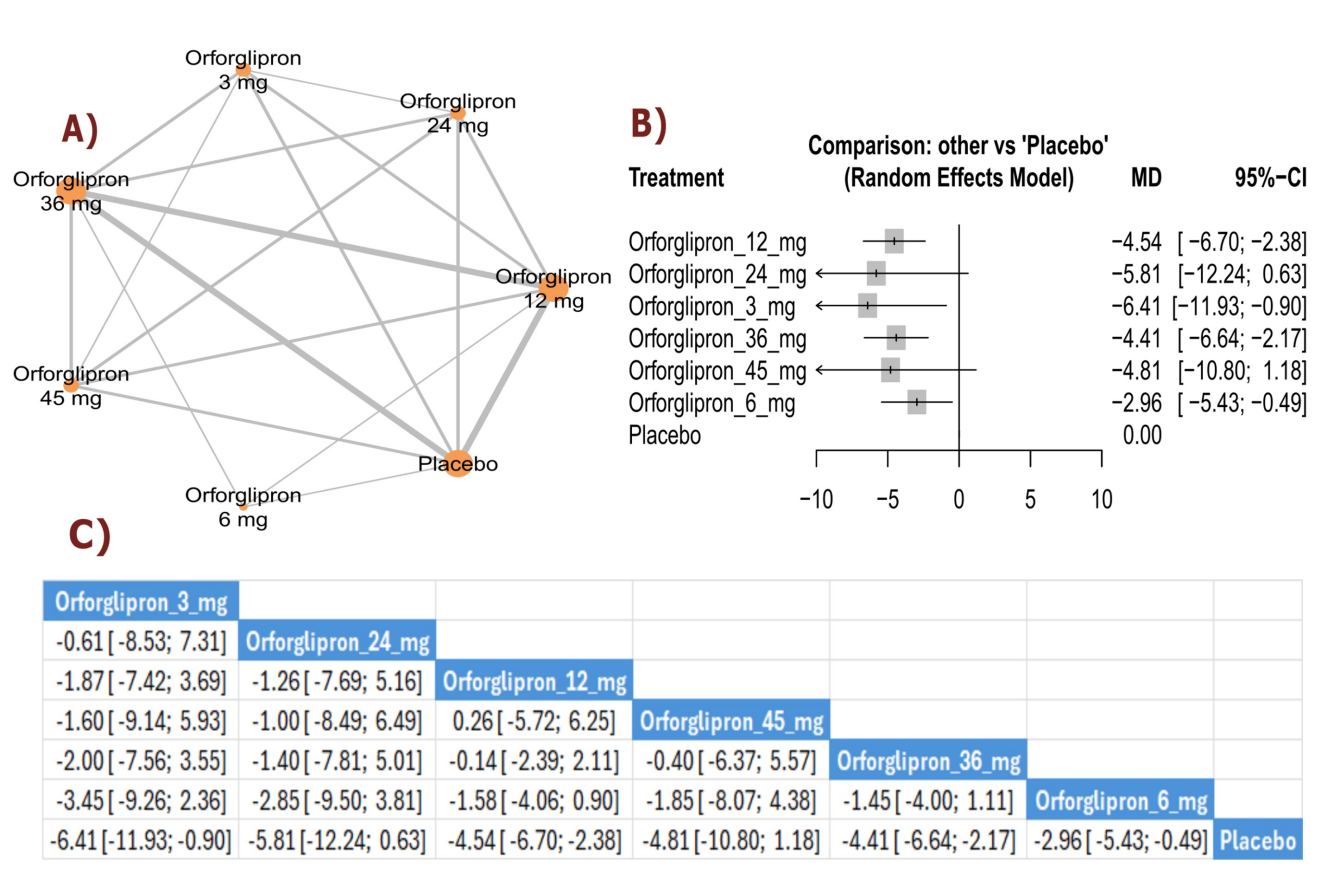
LDL change (%). References: [11,16,25,26,28]. LDL: Low-density lipoprotein.

LDL change (percent): The results demonstrated a significant reduction in LDL cholesterol between orforglipron 3 mg and placebo (MD = -6.41, 95% CI (-11.93; -0.90)), as well as between orforglipron 12 mg and placebo (MD = -4.54, 95% CI (-6.70; -2.38)). The top three treatments for LDL cholesterol reduction, ranked by P-score, were orforglipron 3 mg, orforglipron 24 mg, and orforglipron 12 mg (Figure 12).

Blood Pressure Outcomes

**Figure 13:**
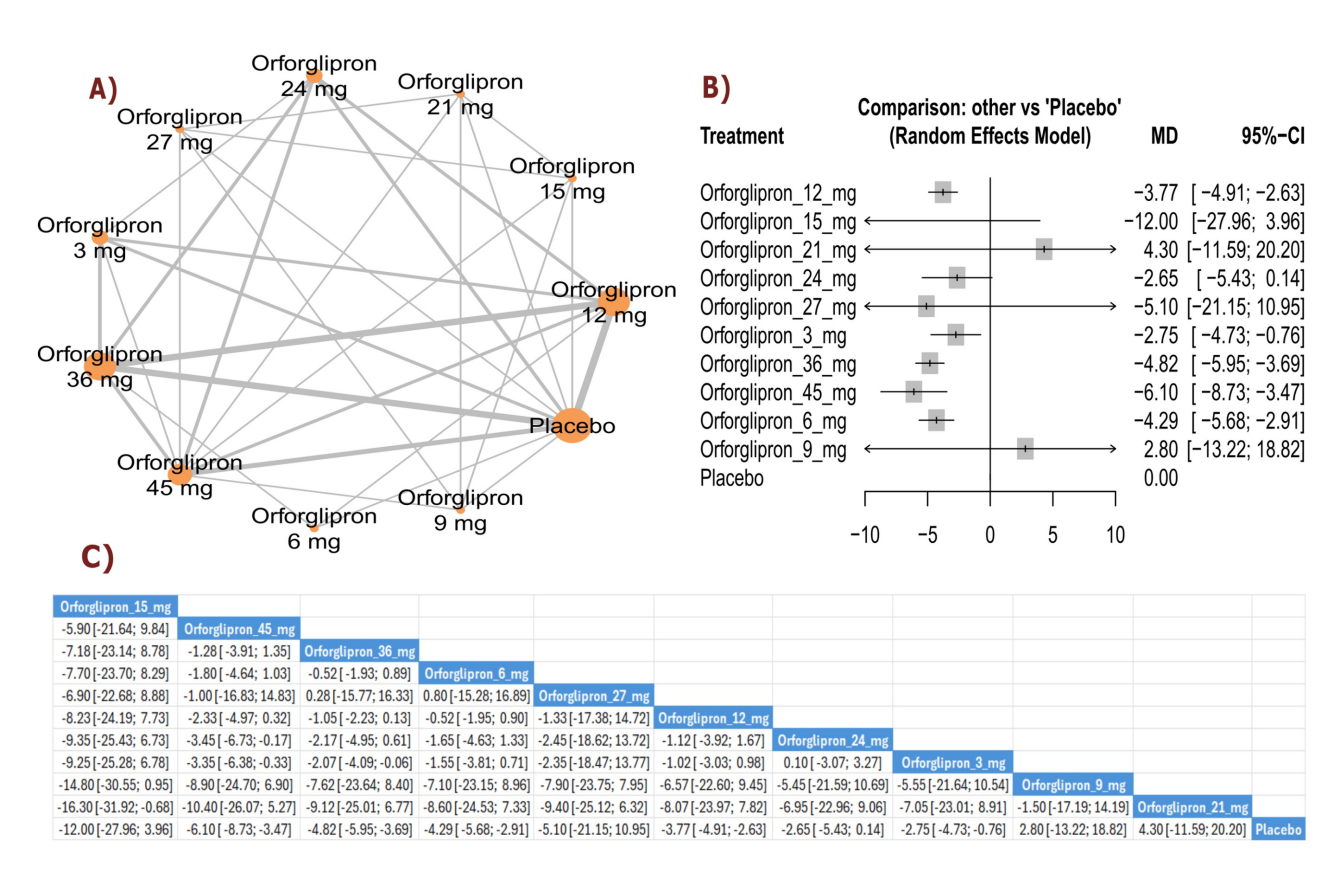
Systolic blood pressure change (mm Hg). References: [11,16,25,26,28].

SBP change (mmHg): The results showed a significant reduction in SBP with orforglipron 6 mg compared with placebo (MD = -4.82, 95% CI (-5.95; -3.69)). Multiple orforglipron doses demonstrated significant SBP reductions compared with placebo. The top three treatments for SBP reduction, ranked by P-score, were orforglipron 15 mg, orforglipron 45 mg, and orforglipron 36 mg (Figure 13).

**Figure 14:**
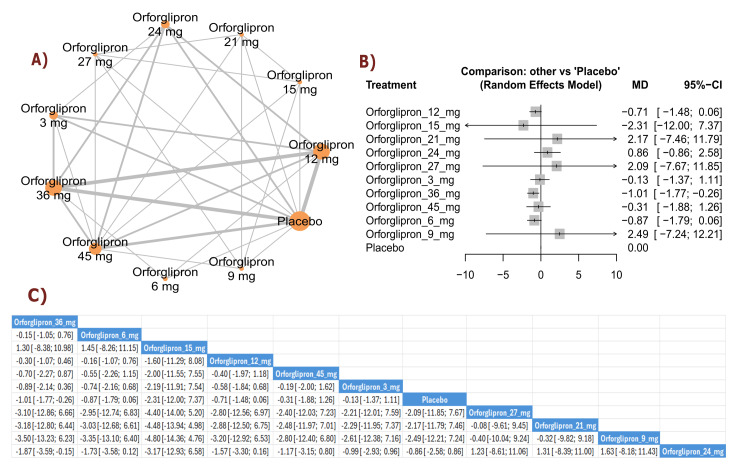
Diastolic blood pressure change (mm Hg). References: [11,16,25,26,28].

DBP change (mmHg): The results demonstrated a significant reduction in DBP between orforglipron 24 mg and placebo (MD = -1.87, 95% CI (-3.59; -0.15)), as well as between orforglipron 36 mg and placebo (MD = -1.01, 95% CI (-1.77; -0.26)). Several orforglipron doses showed significant DBP reductions compared with placebo. The top three treatments for DBP reduction according to P-score were orforglipron 24 mg, followed by orforglipron 36 mg, and orforglipron 6 mg (Figure 14).

Adverse Events (General Safety)

**Figure 15:**
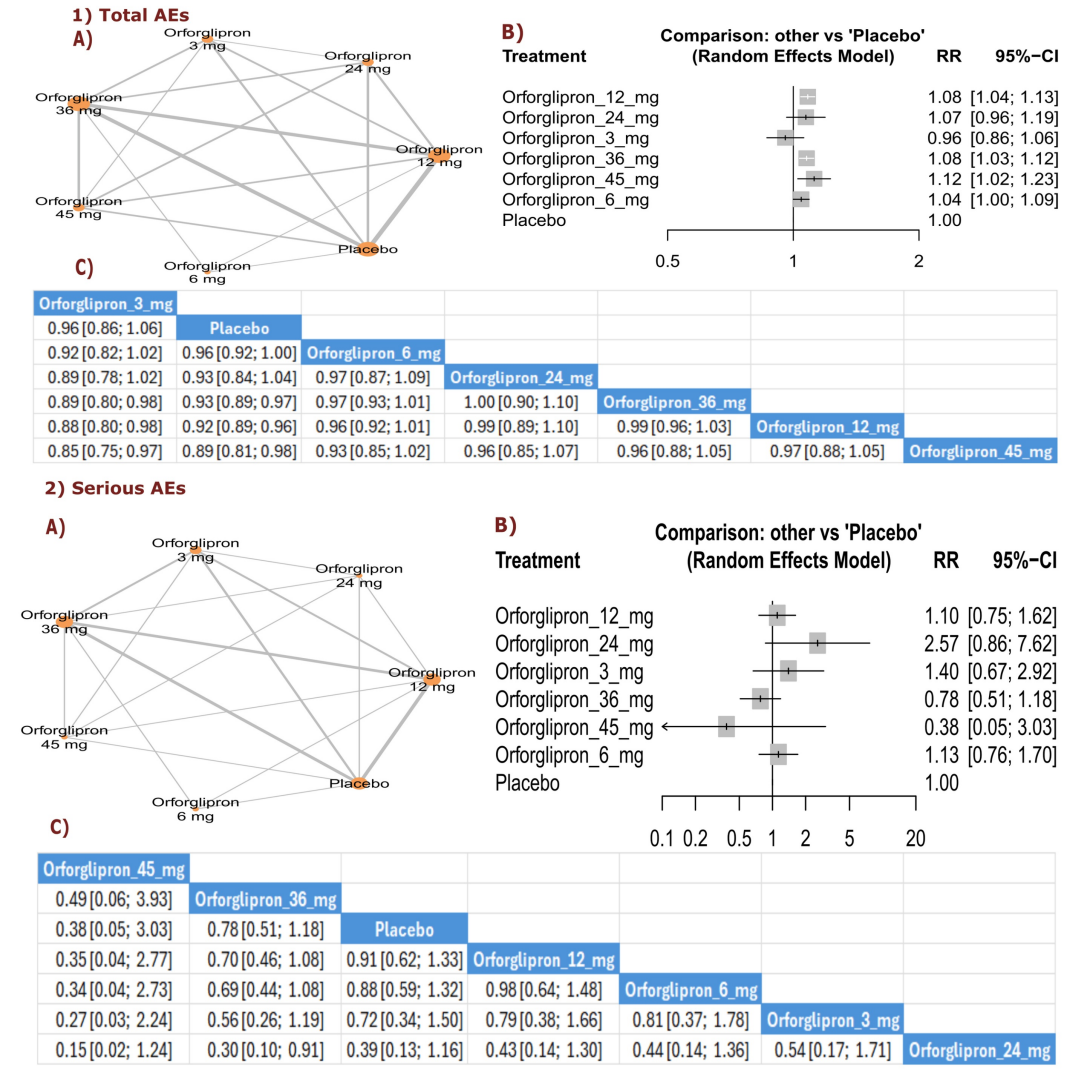
Total adverse events and serious adverse events. References: [11,16,25,26,28].

Total AEs: All orforglipron doses showed RR > 1.00 compared with placebo, indicating higher rates of total adverse events. The results showed significant increases in total adverse events with orforglipron 45 mg compared with placebo (RR = 1.12, 95% CI (1.02; 1.23)) and orforglipron 12 mg compared with placebo (RR = 1.08, 95% CI (1.04; 1.13)). All orforglipron doses showed higher rates of total adverse events than placebo. The three treatments with the highest incidence of total adverse events according to P-score were orforglipron 45 mg, followed by orforglipron 36 mg, and orforglipron 12 mg (Figure 15).

Serious AEs: Serious adverse events were generally lower with orforglipron treatment compared with placebo, with orforglipron 24 mg showing a statistically significant reduction (RR = 0.30, 95% CI (0.10; 0.91)). Most other doses also demonstrated numerically fewer serious adverse events than placebo. The treatments with the lowest incidence of serious adverse events according to P-score were orforglipron 24 mg, orforglipron 45 mg, and orforglipron 36 mg (Figure 15).

**Figure 16:**
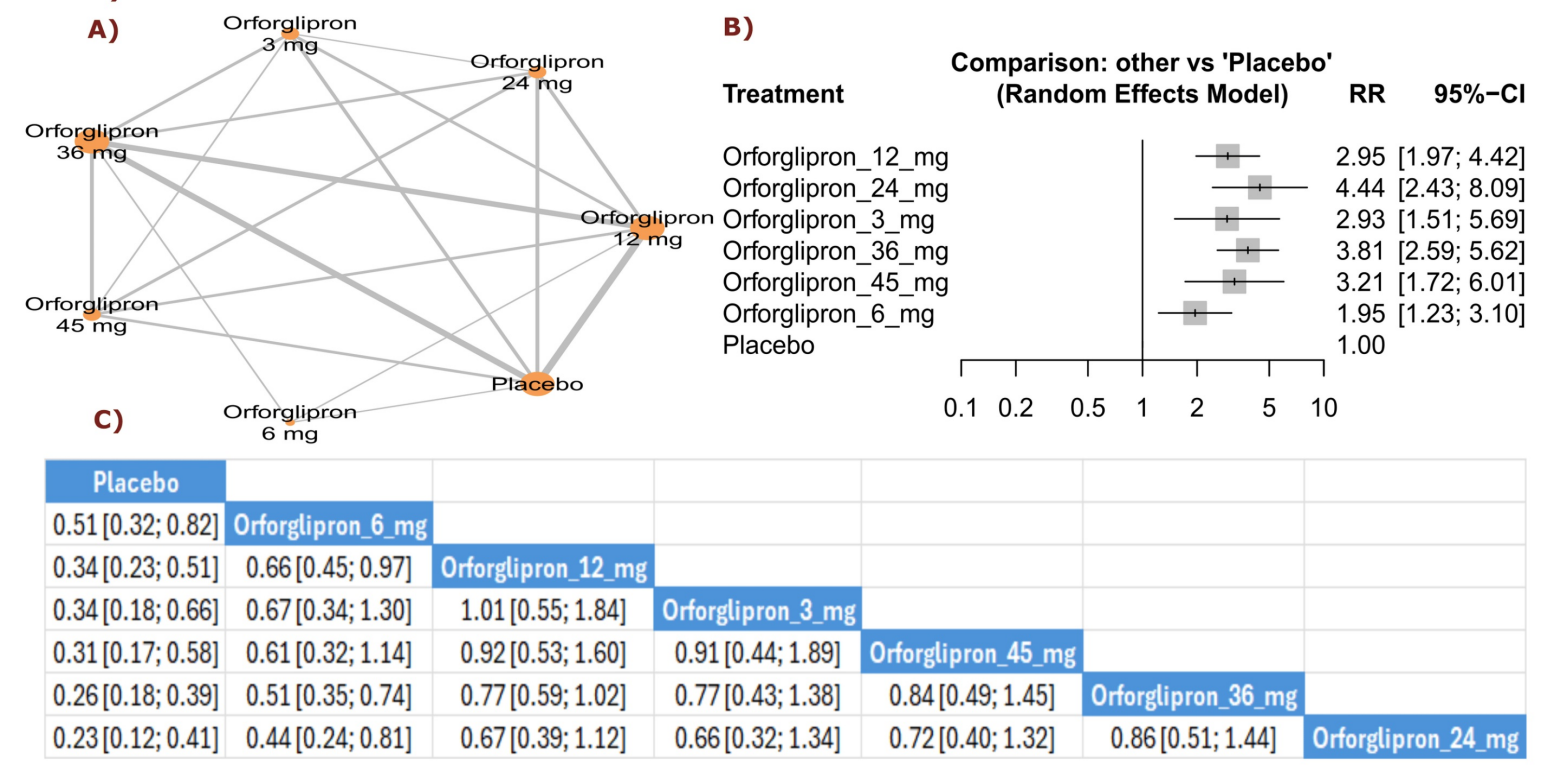
Treatment discontinuation due to adverse events. References: [11,16,25,26,28].

Treatment discontinuation due to AEs: The results revealed that treatment discontinuation due to adverse events was significantly more frequent with all orforglipron doses compared with placebo, with orforglipron 24 mg showing the highest rate (RR = 4.44, 95% CI (2.43; 8.09)), followed by orforglipron 36 mg (RR = 3.81, 95% CI (2.59; 5.62)) and orforglipron 45 mg (RR = 3.21, 95% CI (1.72; 6.01)) (Figure 16).

AEs: Gastrointestinal

**Figure 17:**
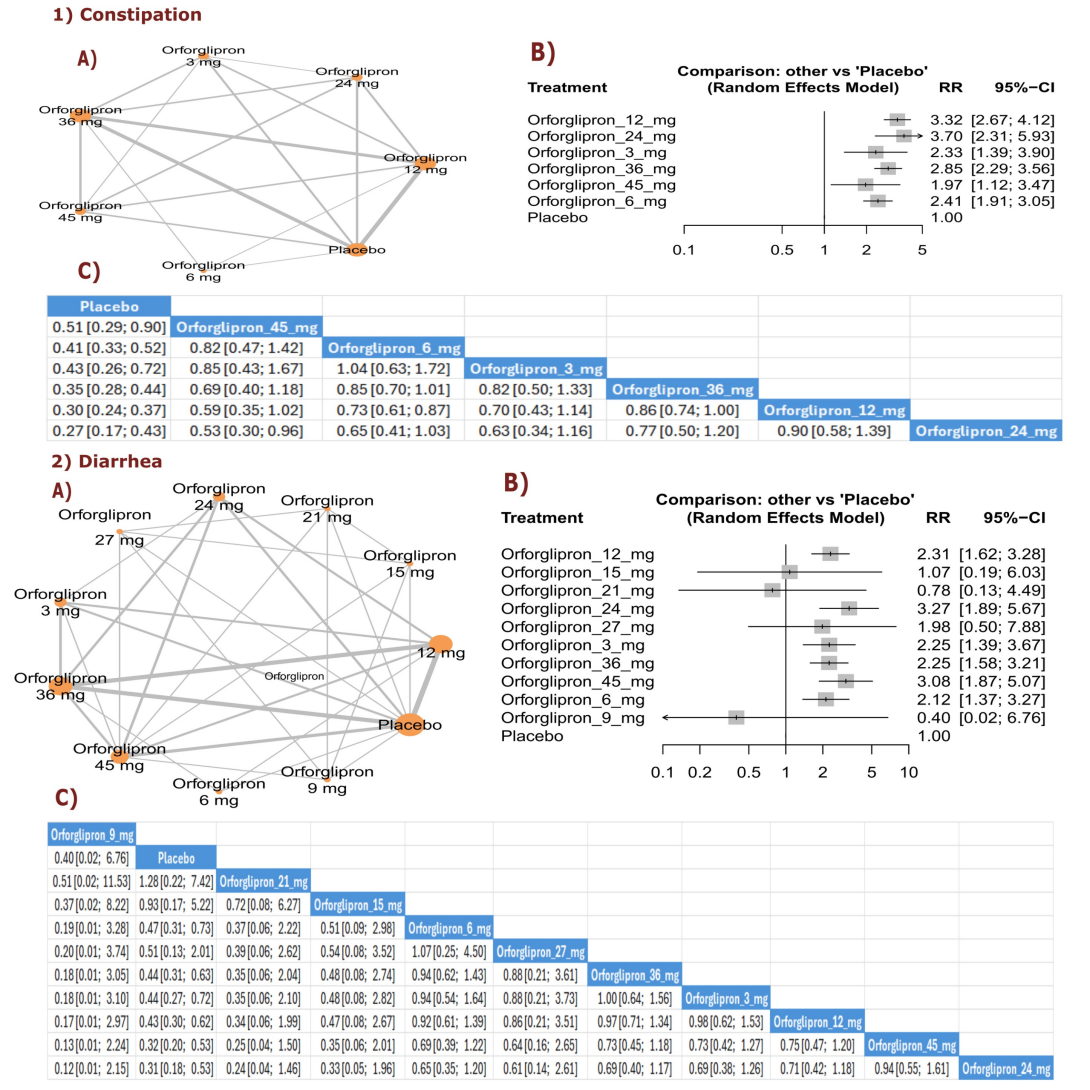
Constipation and diarrhea. References: [11,16,25,26,28].

Constipation: The results demonstrated significant variations in constipation rates. Orforglipron 45 mg showed lower rates than orforglipron 6 mg (RR = 0.47, 95% CI (0.31; 0.73)), while multiple doses showed lower rates than placebo. The three best treatments, ranked by P-score for lower constipation incidence, were orforglipron 24 mg, orforglipron 45 mg, and orforglipron 6 mg (Figure 17).

Diarrhea: The results showed a significant reduction in diarrhea incidence with orforglipron 9 mg compared with placebo (RR = 0.51, 95% CI (0.29; 0.90)). Multiple orforglipron doses demonstrated lower diarrhea rates compared with placebo. The three best treatments, ranked by P-score for lower diarrhea incidence, were orforglipron 21 mg, followed by orforglipron 15 mg, and orforglipron 27 mg (Figure 17).

**Figure 18:**
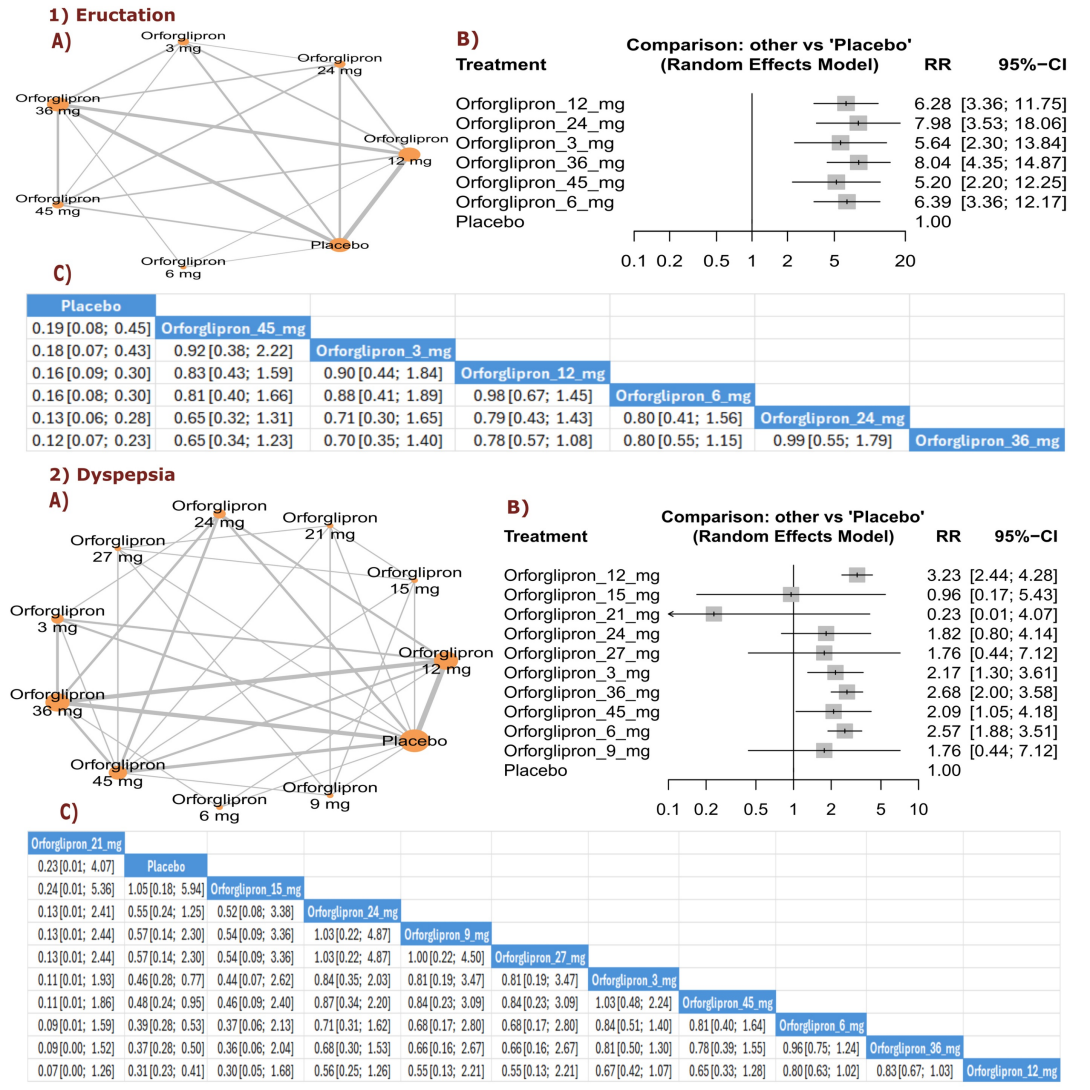
Dyspepsia and eructation. References: [11,16,25,26,28].

Dyspepsia and eructation: The results demonstrated significant reductions in dyspepsia with orforglipron 45 mg compared with placebo (RR = 0.22, 95% CI (0.09; 0.54)), as well as with orforglipron 24 mg compared with placebo (RR = 0.26, 95% CI (0.08; 0.86)). Most orforglipron doses showed significantly lower dyspepsia rates compared with placebo. The three best treatments, ranked by P-score for lower dyspepsia incidence, were orforglipron 45 mg, followed by orforglipron 27 mg, and orforglipron 24 mg. No significant differences were found among the available treatments in terms of eructation incidence (Figure 18).

**Figure 19:**
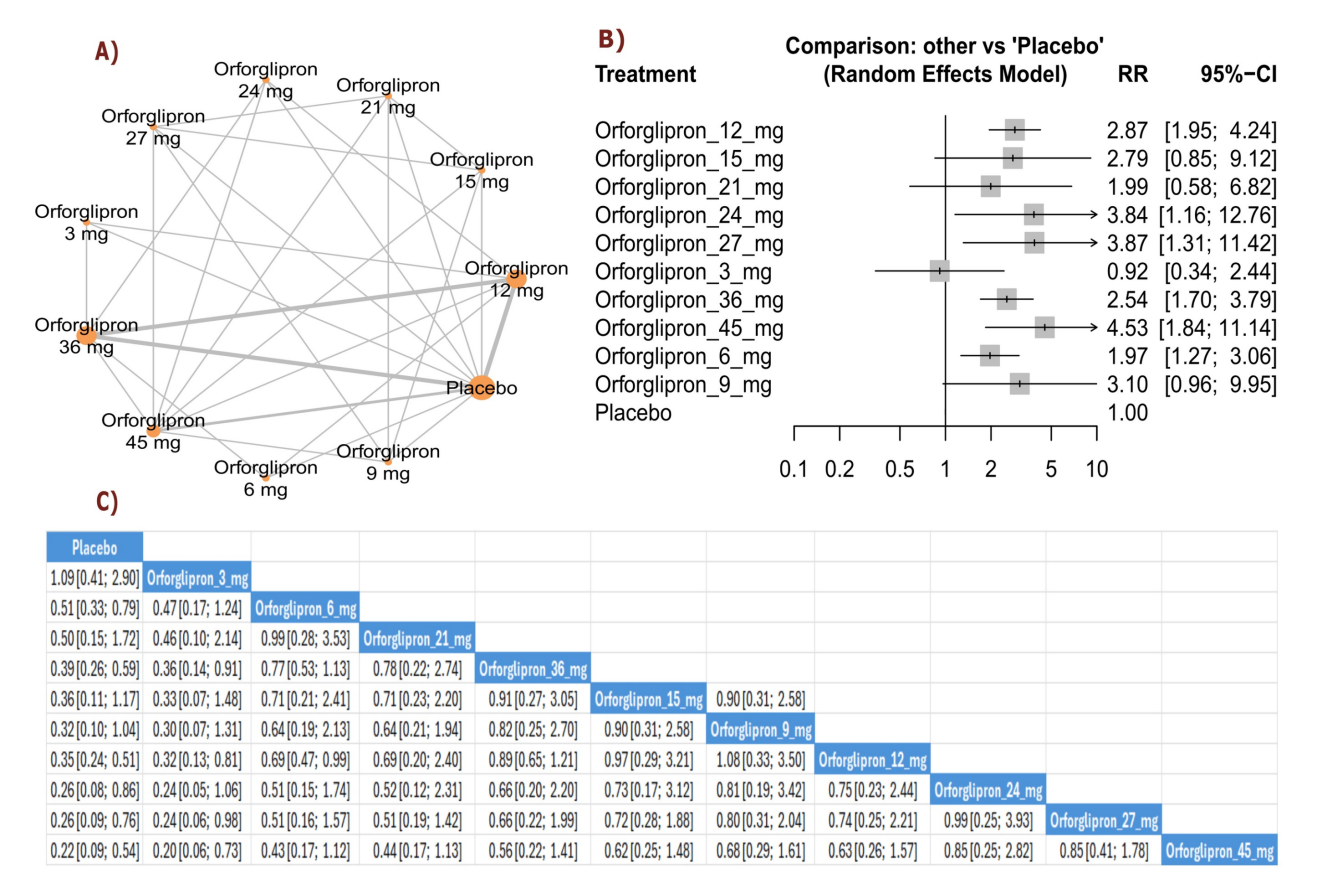
Decreased appetite. References: [11,16,25,26,28].

Decreased appetite: The results demonstrated significant increases in appetite suppression with orforglipron 45 mg compared with placebo (RR = 4.53, 95% CI (1.84; 11.14)), as well as with orforglipron 24 mg compared with placebo (RR = 3.84, 95% CI (1.16; 12.76)). All orforglipron doses showed significantly higher rates of appetite suppression compared with placebo. The three most effective treatments for inducing appetite suppression according to P-score were orforglipron 45 mg, followed by orforglipron 27 mg, and orforglipron 24 mg (Figure 19).

**Figure 20:**
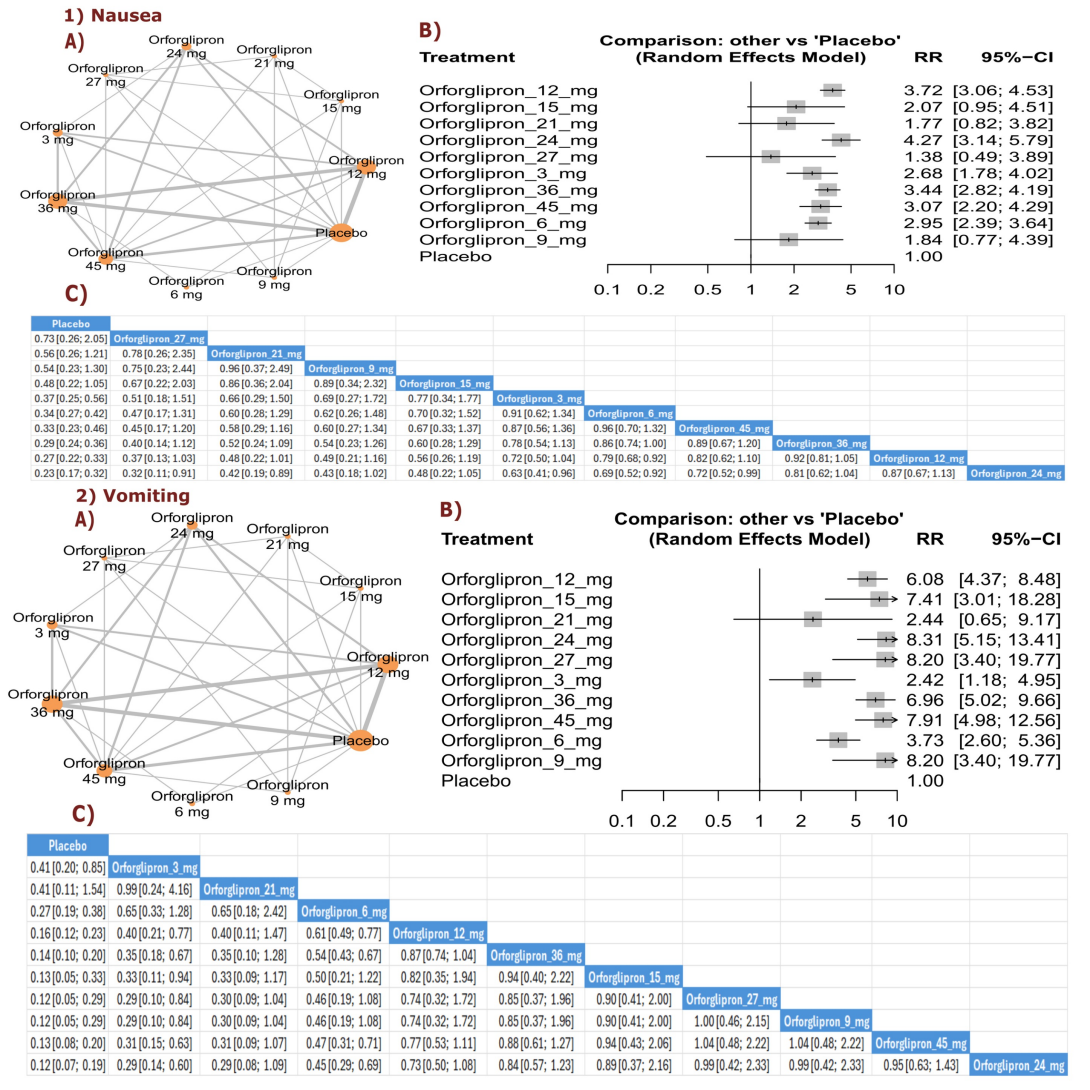
Nausea and vomiting. References: [11,16,25,26,28].

Nausea: The results demonstrated significant reductions in nausea with orforglipron 12 mg compared with placebo (RR = 0.16, 95% CI (0.12; 0.23)), as well as with orforglipron 36 mg compared with placebo (RR = 0.14, 95% CI (0.10; 0.20)). All orforglipron doses showed significantly lower nausea rates compared with placebo. The top three treatments for lower nausea incidence according to P-score were orforglipron 15 mg, followed by orforglipron 27 mg, and orforglipron 9 mg (Figure 20).

Vomiting: The results showed significant reductions in vomiting with orforglipron 12 mg compared with placebo (RR = 0.16, 95% CI (0.09; 0.30)), as well as with orforglipron 36 mg compared with placebo (RR = 0.12, 95% CI (0.07; 0.23)). Most orforglipron doses showed significantly lower vomiting rates than placebo. The top three treatments for lower vomiting incidence according to P-score were orforglipron 36 mg, followed by orforglipron 12 mg, and orforglipron 24 mg (Figure 20).

**Figure 21:**
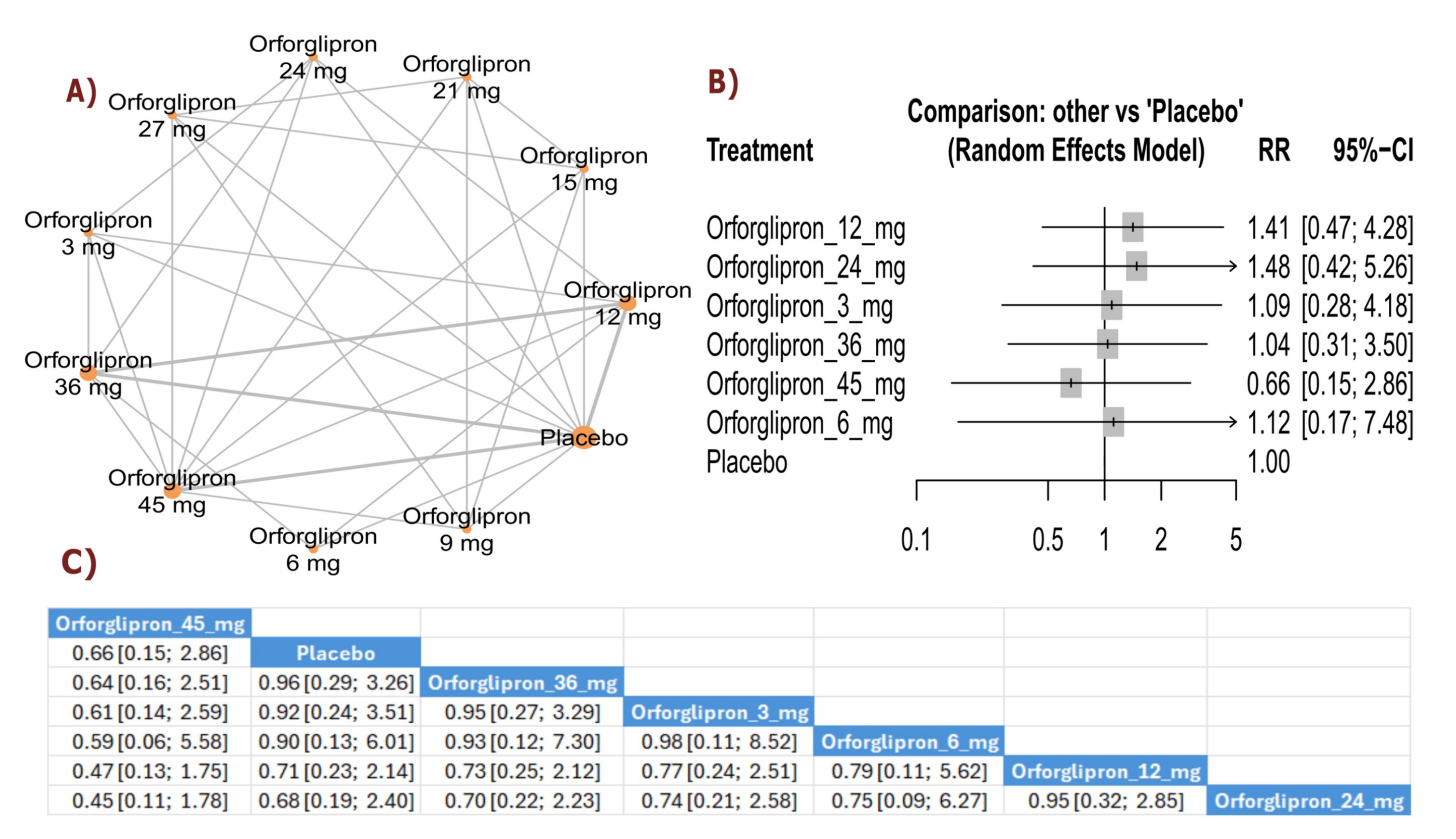
Cardiac disorders. References: [11,16,25,26,28].

Cardiac disorders: No significant differences were found among the available treatments in terms of cardiac disorder incidence (Figure 21).

Systematic Review

Exploratory analyses from a Phase 2 trial (Rosenstock 2025) evaluated orforglipron’s effects on biomarkers of β-cell function and insulin sensitivity in 378 participants with T2DM over 26 weeks. Orforglipron doses of 12 mg or higher significantly improved β-cell function, with HOMA-B increasing by 123% (C-peptide) and 132% (insulin) by week 4 and sustained through week 26, exceeding improvements observed with dulaglutide 1.5 mg. The proinsulin/insulin ratio decreased by 26-55% across doses, indicating enhanced β-cell efficiency. Insulin sensitivity markers also improved, with HOMA-IR (insulin) decreasing by up to 23% by week 26, IGFBP2 increasing by 29-60%, and fasting glucagon reduced by up to 38.5% at the 45 mg dose. These mechanistic findings suggest orforglipron enhances pancreatic β-cell function and insulin sensitivity through pathways consistent with established injectable GLP-1RAs [27].

Discussion

This network meta-analysis evaluated the efficacy and safety of orforglipron (3-45 mg) across six RCTs in patients with type 2 diabetes and obesity. Orforglipron demonstrated significant improvements in weight reduction, glycemic control, lipid profile, and blood pressure compared with placebo. Efficacy was generally dose-dependent, with higher doses, particularly 45 mg, showing superior performance for weight-related outcomes and HbA1c reduction. Interestingly, mid-range doses (24-36 mg) achieved optimal results for triglyceride reduction and blood pressure control. Regarding safety, all orforglipron doses were associated with higher rates of total adverse events compared with placebo, representing a significant clinical consideration. Notably, treatment discontinuation due to adverse events was substantially higher across all active doses, particularly at 24-45 mg, which may impact long-term treatment adherence and real-world effectiveness. While serious adverse events were numerically lower than placebo, the higher discontinuation rates represent a key limitation that must be carefully weighed against the metabolic benefits. These safety findings underscore the importance of individualized dose titration and close monitoring during orforglipron therapy.

Clinically meaningful weight reduction of 5-10% has been associated with improvements in cardiometabolic risk factors, while weight loss exceeding 15% confers additional cardiovascular benefits [17]. Our findings demonstrate that orforglipron not only achieved these therapeutic weight loss thresholds but also produced concurrent improvements in key cardiovascular risk factors, including significant reductions in blood pressure, triglycerides, and LDL cholesterol, supporting its potential role in comprehensive cardiometabolic risk reduction. In the context of current American Diabetes Association and European Association for the Study of Diabetes guidelines for managing hyperglycemia in T2D, which emphasize the importance of glucose-lowering agents with weight-reducing properties and highlight the value of simplified treatment regimens to improve adherence and persistence, these efficacy findings with orforglipron are clinically meaningful [29].

Orforglipron functions as a biased GLP-1 receptor agonist, preferentially stimulating cyclic adenosine monophosphate (cAMP) accumulation while minimizing β-arrestin recruitment [17]. This selectivity is mechanistically significant, as β-arrestin mediates receptor desensitization and internalization [19]. By limiting β-arrestin engagement, orforglipron may sustain receptor signaling and prolong therapeutic effects, potentially explaining the robust metabolic improvements observed in our analysis. This biased agonism mechanism parallels that of tirzepatide, a dual GLP-1/GIP receptor agonist that has demonstrated superior efficacy compared with selective GLP-1 receptor agonists in clinical trials [30,31].

Tirzepatide’s enhanced metabolic effects have been partially attributed to preferential cAMP signaling with reduced β-arrestin recruitment at the GLP-1 receptor, enabling more durable receptor activation. The shared mechanistic profile between orforglipron and tirzepatide suggests that biased agonism may represent an important determinant of GLP-1 receptor agonist efficacy. However, direct comparative studies are needed to establish whether orforglipron achieves equivalent clinical benefits to tirzepatide and to fully elucidate the therapeutic implications of this signaling bias [32].

Poor oral absorption of peptide-based therapeutics, including incretin medications, significantly limits their use as oral formulations. Existing incretin therapies are predominantly administered by injection, except for oral semaglutide, which has extremely low bioavailability (0.4%-1.0%) and requires co-formulation with absorption enhancers, necessitating administration under strict fasting conditions with minimal water intake [33]. These requirements may compromise patient adherence and limit the practical utility of oral semaglutide in real-world settings. The development of non-peptide GLP-1 receptor agonists addresses these limitations by substantially improving oral bioavailability. Orforglipron, as a chemically synthesized, non-peptide oral GLP-1 receptor agonist, achieves considerably higher bioavailability (20%-40% based on preclinical data) compared with oral semaglutide [32]. Critically, orforglipron can be administered once daily without dietary restrictions or fasting requirements, while producing comparable reductions in HbA1c and BW to those observed with oral semaglutide in Phase 1 trials [16]. This combination of improved bioavailability, simplified dosing, and robust efficacy positions orforglipron as a potentially transformative advancement in oral incretin therapy, with the potential to significantly enhance treatment adherence and expand access to GLP-1 receptor agonist therapy.

Orforglipron’s influence on safety laboratory parameters, particularly pancreatic enzymes and calcitonin levels, mirrored the patterns observed with other GLP-1 receptor agonists such as semaglutide and dulaglutide [34,35]. These findings suggest a class effect rather than drug-specific aberrations. The consistency across different GLP-1 receptor agonists reinforces the importance of routine monitoring of these parameters during treatment, while also providing reassurance regarding orforglipron’s safety profile within this therapeutic class [36].

The therapeutic landscape of GLP-1 receptor agonists continues to expand, with novel agents demonstrating robust efficacy in obesity management. Recent meta-analyses of survodutide and retatrutide have shown significant BW reductions and improvements in metabolic parameters, consistent with the established benefits of this drug class. Our findings with orforglipron align with these emerging data, demonstrating that non-peptide GLP-1 receptor agonists can achieve comparable metabolic benefits while offering practical advantages in oral bioavailability and simplified dosing. The consistency of weight reduction and glycemic control across different GLP-1 receptor agonist formulations reinforces the class effect of these therapeutic agents and supports orforglipron’s potential role in comprehensive cardiometabolic risk management [37,38]. Additionally, orforglipron may have similar adjuvant effects to those observed with GLP-1 receptor agonists in improving healing and recovery in different surgical settings [39].

This study has several strengths, as it is the first meta-analysis to compare the safety and effectiveness of orforglipron across multiple doses. Second, the inclusion of only RCTs ensured the quality of the evidence, as RCTs are the gold standard for evidence synthesis. Third, the network meta-analysis approach enabled simultaneous comparison of multiple doses that were not directly compared in head-to-head trials, providing a clinically valuable ranking of treatment options. Fourth, the analysis evaluated a comprehensive range of outcomes encompassing glycemic control, weight reduction, cardiovascular risk factors, and detailed safety profiles, offering a holistic assessment of orforglipron’s therapeutic profile. Fifth, including studies with diverse patient populations across multiple countries enhances the generalizability of the findings.

Several limitations warrant consideration when interpreting these findings. First, the relatively short duration of the included studies (12-72 weeks) limits assessment of long-term efficacy and safety. Second, three of the six included studies raised concerns in domains related to randomization and missing outcome data. Third, heterogeneity in baseline characteristics, including variations in diabetes duration, metformin use, and obesity severity, may have affected treatment responses. Fourth, the paradoxical reduction in gastrointestinal AEs compared with placebo raises questions about the consistency of outcome reporting across trials. Finally, the higher treatment discontinuation rates observed with orforglipron may reflect the trial setting and could differ in real-world clinical practice.

Several areas warrant further investigation. Long-term studies are essential to evaluate sustained efficacy, weight-loss durability, and cardiovascular outcomes. Dedicated cardiovascular outcome trials and comparative effectiveness studies with established GLP-1 receptor agonists are needed. Research on optimal dosing strategies, diverse populations, and real-world effectiveness would enhance clinical applicability. Additionally, mechanistic studies, pharmacoeconomic analyses, and investigations into combination therapy may optimize treatment approaches.

## Conclusions

Orforglipron is particularly suitable for patients who prefer oral therapy over injectable GLP-1 receptor agonists. Orforglipron demonstrated significant dose-dependent improvements, with high doses (45 mg) recommended for patients requiring maximum weight loss, poorly controlled T2DM, and aggressive cardiometabolic risk management. Mid-range doses (24-36 mg) are better suited for patients prioritizing lipid management and blood pressure control while seeking improved tolerability and lower discontinuation risk. Low doses (3-12 mg) may be considered for patients initiating therapy, those with mild metabolic dysfunction, or individuals particularly sensitive to GLP-1 receptor agonist side effects, allowing gradual dose escalation based on individual response and tolerability. The combination of improved oral bioavailability, simplified once-daily dosing without dietary restrictions, and flexible dose titration options positions orforglipron as a promising advancement in oral incretin therapy. However, careful monitoring for treatment tolerability remains essential given higher discontinuation rates at elevated doses. Future research should focus on long-term cardiovascular outcome trials, head-to-head comparative studies with established GLP-1 receptor agonists, optimal dose titration strategies, and real-world effectiveness studies in diverse patient populations to establish definitive patient selection criteria and therapeutic positioning within the evolving landscape of diabetes and obesity management.

## Appendices

Appendix 1 

**Table 3:** Full detailed search strategy.

Database	Search query	Results
PubMed	(Orforglipron OR LY3502970 OR LY-3502970 OR V6G OR OWL833) AND (Obesity OR Obese OR Overweight OR adiposity OR corpulence OR plumpness OR "excess body weight" OR Diabetes OR Diabetic OR MODY OR IDDM)	48
Scopus	TITLE-ABS-KEY ( ( Orforglipron OR LY3502970 OR LY-3502970 OR V6G OR OWL833 ) AND ( Obesity OR Obese OR Overweight OR adiposity OR corpulence OR plumpness OR "excess body weight" OR Diabetes OR Diabetic OR MODY OR IDDM ) )	103
Web of Science	TS=((Orforglipron OR LY3502970 OR LY-3502970 OR V6G OR OWL833) AND (Obesity OR Obese OR Overweight OR adiposity OR corpulence OR plumpness OR "excess body weight" OR Diabetes OR Diabetic OR MODY OR IDDM))	51
Cochrane Library	((Orforglipron OR LY3502970 OR LY-3502970 OR V6G OR OWL833) AND (Obesity OR Obese OR Overweight OR adiposity OR corpulence OR plumpness OR "excess body weight" OR Diabetes OR Diabetic OR MODY OR IDDM)):ti,ab,kw	60
Embase	(orforglipron:ab,ti OR ly3502970:ab,ti OR ly-3502970:ab,ti OR 'ly 3502970':ab,ti OR v6g:ab,ti OR owl833:ab,ti) AND (obesity:ab,ti OR obese:ab,ti OR overweight:ab,ti OR adiposity:ab,ti OR corpulence:ab,ti OR plumpness:ab,ti OR 'excess body weight':ab,ti OR diabetes:ab,ti OR diabetic:ab,ti OR mody:ab,ti OR iddm:ab,ti)	77
